# Genetic Diversity, Chemical Constituents, and Bioactivities of *Maerua siamensis* from Thailand

**DOI:** 10.3390/plants13233359

**Published:** 2024-11-29

**Authors:** Natthawadi Wongthet, Markus Bacher, Mara Krenn, Apichart Jai-aree, Thomas Rosenau, Patsakorn Tiwutanon, Nopparat Anantaprayoon, Srunya Vajrodaya, Lothar Brecker, Johann Schinnerl, Ekaphan Kraichak

**Affiliations:** 1 Department of Botany, Faculty of Science, Kasetsart University, Bangkok 10900, Thailand; natthawadi.won@ku.th (N.W.); patsakorn.a@ku.th (P.T.); nopparat.ana@ku.th (N.A.); fscisyv@ku.ac.th (S.V.); 2 Department of Chemistry, Institute of Chemistry of Renewable Resources, University of Natural Resources and Life Sciences (BOKU), A-3430 Tulln, Austria; markus.bacher@boku.ac.at (M.B.); thomas.rosenau@boku.ac.at (T.R.); 3 Department of Botany and Biodiversity Research, University of Vienna, Rennweg 14, A-1030 Vienna, Austria; mara.krenn@univie.ac.at; 4 Department of Human and Community Resource Development, Faculty of Education and Development Sciences, Kamphaeng Saen Campus, Kasetsart University, Nakhon Pathom 73140, Thailand; feduacj@ku.ac.th; 5 Department of Organic Chemistry, University of Vienna, Währinger Strasse 38, A-1090 Vienna, Austria; lothar.brecker@univie.ac.at; 6 Biodiversity Center, Kasetsart University, Bangkok 10900, Thailand

**Keywords:** Capparaceae, genetic diversity, maeruamide, maeruaoside, phytochemistry

## Abstract

The genus *Maerua* (Capparaceae) comprises 85 woody species distributed across the paleotropics, with some species used in traditional medicine. This study investigated the phylogenetic placement, genetic diversity, and phytochemical composition of *Maerua siamensis*, a species native to Indochina. Phylogenetic analyses using *matK* and *rbcL* markers confirmed the monophyly of the *M. siamensis* specimens collected from Thailand. Population genetic analyses revealed low genetic diversity across the sampled populations, suggesting purifying selection or recent demographic events. Phytochemical investigations of the leaf extracts led to the isolation and identification of 12 compounds, including two novel structures: maeruamide and maeruaoside. The isolated compounds encompass simple phenolics, l-proline-derived betaines, and flavonoid glycosides. Bioactivity assays revealed moderate antioxidant activity of the crude extract (EC_50_ = 106 μg mL^−1^), but no significant insecticidal or nematocidal effects were observed. This study provides new insights into the genetic diversity, phytochemistry, and potential bioactivities of *M. siamensis*, contributing to our understanding of this understudied species and its ethnomedicinal applications.

## 1. Introduction

The palaeotropical distributed genus *Maerua* (Capparaceae; order Brassicales) comprises 85 accepted woody species, of which some species have been described within the last twenty years [1,2]. Various *Maerua* species have been the subject of phytochemical investigations because these plants are used in traditional medicine [3,4]. People in West Africa also use parts of *Maerua* species, like leaves of *M*. *angolensis* DC., *M*. *crassifolia* Forssk., and *M*. *subcordata* (Gilg) DeWolf to treat psychosis, ecthyma, epilepsy, diarrhea, and dysentery, malaria and other infectious diseases, and allergic and gastrointestinal disorders, as well as to relieve pain [3,5,6]. Several phytochemical studies of this genus have been conducted since 1995. Megastigmane glucoside and quercetin-3-*O*-β-d-galactoside have been reported in the aerial parts of *M. crassifolia* [7]. Aminoguanidine derivates were extracted from leaves of *M. edulis* (Gilg & Gilg-Ben.) DeWolf [4]. Indole alkaloids have been reported in the roots of *M*. *siamensis* (Kurz) Pax [8]. Indole nitrile glycosides have also been reported in the leaves of this species [9]. Glucosinolates, as expected from this genus due to its close relationship to *Capparis*, were confirmed for the species *M*. *angolensis*, *M*. *crassiflora*, and *M*. *triphylla* A.Rich. Methyl- and indol-3-yl-methyl glucosinolates have been identified as major glucosinolate components [10]. Prior research has primarily examined African species and their chemical components. There is a significant gap in the knowledge regarding Asian species and genetic variations that may influence their phytochemical profiles.

**Figure 1:**
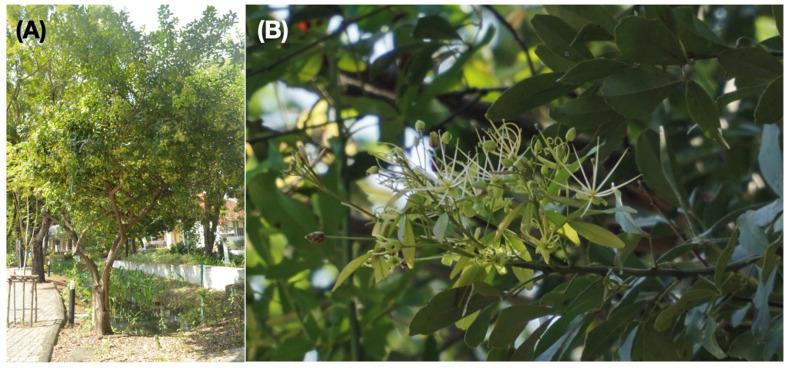
*Maerua siamensis* from Thailand: (**A**) tree habitat and (**B**) flowers and leaves. Photo: P. Tiwutanon.

The investigated species, *Maerua siamensis* (Kurz) Pax., is native to Indochina (Figure 1) and is distributed throughout the central, northeastern, and western regions of Thailand, Cambodia, and Vietnam [11]. Native people from Thailand, such as the Mien people in the Chat Nai province, central Thailand, use decoctions of leaves, stems, and roots of *M*. *siamensis* to relieve headaches and toothache, reduce swelling, and for other health-related purposes [12,13,14,15]. *Maerua siamensis* stands out as one of the only two Asian species in the genus, with the other being *M. koratensis*, also described from Thailand. This unique status has led to uncertainties in its phylogenetic position and taxonomic classification. The limited geographical range of *M. siamensis* also raises concerns regarding its genetic diversity. Species with restricted distributions typically exhibit reduced genetic variability, which can increase their vulnerability to extinction [16,17].

This study aimed to provide a comprehensive understanding of *Maerua siamensis* by analyzing its phylogenetic position, genetic diversity, and phytochemical composition. We collected *M. siamensis* specimens from various locations in Thailand for both genetic and phytochemical investigation. Phylogenetic reconstruction and population genetic analyses were conducted using chloroplast marker sequences (*matK* and *rbcL*) to elucidate the evolutionary relationships of the species and assess their genetic diversity. Concurrently, chromatographic techniques were employed to isolate and characterize the phytochemical components of the leaf extracts. These extracts were subsequently evaluated for their potential bioactivities, including antioxidant, insecticidal, and nematicidal effects. This multifaceted approach can contribute to the fundamental understanding of this ethnobotanically significant, yet understudied species, as well as its potential for medicinal applications.

## 2. Results and Discussion

### 2.1. Phylogenetic Placement

**Figure 2:**
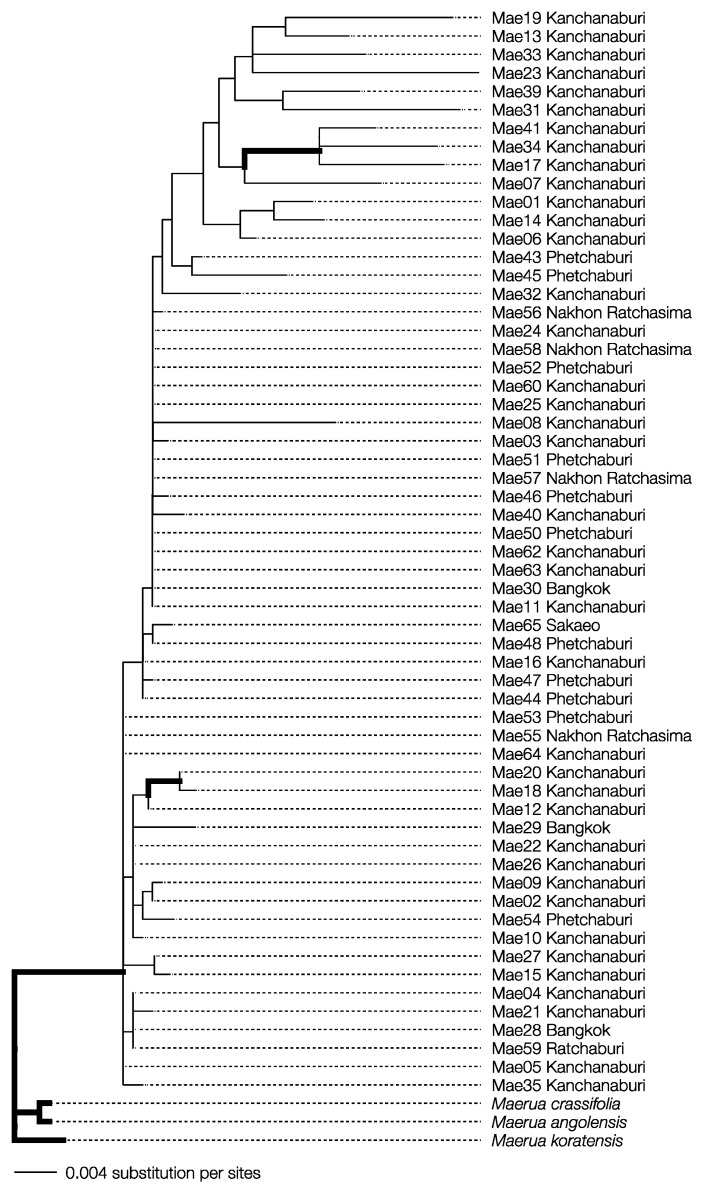
Bayesian inference phylogenetic reconstruction from concatenated dataset alignment of *Maerua siamensis* specimens from Thailand with *M. crassifolia*, *M. koratensis*, and *M. angloensis* as outgroups. Bold branches indicate strong support values from the ML bootstrap values (≥60) and Bayesian posterior probability (≥0.95).

The specimens collected were morphologically identified as *M. siamensis*. The final aligned sequences for *matK* and *rbcL* were 742 and 536 bp long, respectively, with the combined dataset totaling 1278 bp. The final maximum likelihood (ML) and Bayesian inference (BI) analyses produced likelihood scores of −2702.633 and −2756.889, respectively. Both phylogenetic methods showed that all *M. siamensis* samples formed a monophyletic group distinct from their congeneric species with strong statistical support (Figure 2; ML bootstrap support ≥ 60, Bayesian posterior probability ≥ 0.95). These findings confirm that the collected specimens were indeed *Maerua siamensis*.

### 2.2. Population Genetic Structure

The genetic diversity of *M. siamensis* varied slightly among the sampled populations (Table 1). Overall, individuals in Thailand demonstrated low genetic diversity, as evidenced by the low nucleotide diversity. The highest diversity was observed in the Kanchanaburi (KA) and Phetchaburi (PB) populations, which is likely attributable to their larger sample sizes. All individuals from the Nakhon Ratchasima population (NR) possessed identical sequences, which resulted in zero diversity. This is presumably a consequence of individuals being closely related. Despite having the smallest number of individuals, the Bangkok Population (BKK) exhibited some genetic diversity, potentially because these trees were planted as ornamentals with origins from multiple locations. Two large populations in KA and PB, as well as the overall population, exhibited significantly negative Tajima’s D values, suggesting similar evolutionary forces acting on the population, likely purifying selection, or a recent demographic event [16].

**Figure 3:**
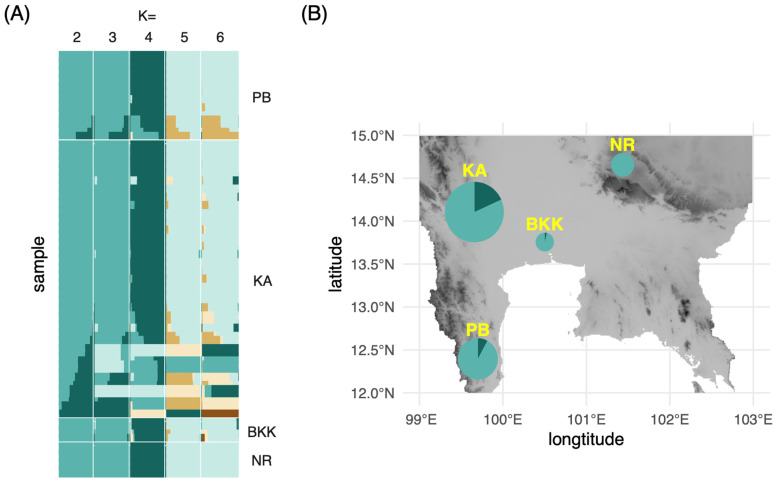
(**A**) Admixture plots from the *matK* and *rbcL* concatenated alignment of *Maerua siamensis* at K = 2–6; (**B**) Proportion of each admixture group at K = 2 on a topographic relief map of Central Thailand. The size of the pie chart corresponds to the number of specimens collected from each province. PB, Petchburi; KA, Kanchanburi; BKK, Bangkok; NR, Nakhon Rachasima.

The individual ancestry coefficients of each *M. siamensis* specimen were estimated under scenarios of K = 1–6 using the concatenated dataset of *matK* and *rbcL* (Figure 3A). The lowest cross-entropy value was at 0.125 in the scenario of K = 2. Consequently, two ancestral populations were selected for the visualization of haplotype frequencies on the map (Figure 3B). The observed low number of ancestral populations was consistent with the significantly negative values of Tajima’s D, indicating purifying selection or selection sweep on populations of these plants. Kanchanaburi (KA) and Phetchaburi (PB) exhibited greater haplotype diversity than the other two provinces, potentially because they contained more sampled individuals. These two provinces are also the type locality from which *M. siamensis* was originally described as *Niebuhria siamensis* [18]. The population in Nakhon Ratchasima (NR) demonstrated the lowest diversity from one haplotype because the sequences from this population were identical. This is potentially due to the small sample size, but is also possibly due to a founder effect from the ancestral population established in this location from the center of diversity in the west. Although a recent evaluation of extinction risks [17] classified *M. siamensis* as “not threatened”, the species exhibits a reduced level of genetic variability, which aligns with the patterns observed in plant populations that are either small or face a high likelihood of extinction [19,20]. It should also be noted that our study only analyzed plastid markers in a limited number of samples. A higher level of genetic variation can be recovered by including nuclear DNA data from more plant samples.

### 2.3. Phytochemical Analyses

**Figure 4:**
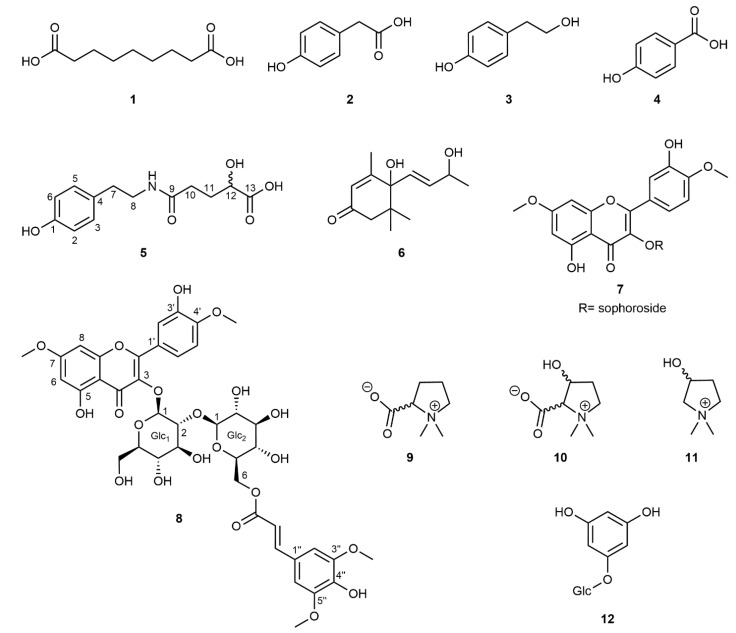
Structural formulae of compounds isolated from *Maerua siamensis*. (**1**) Azelaic acid; (**2**) *para*-hydroxyphenylacetic acid; (**3**) 2-(4-hydroxyphenyl)ethanol; (**4**) *para*-hydroxybenzoic acid; (**5**) maeruamide; (**6**) blumenol A; (**7**) tamarixetin-3-*O*-β-sophoroside-7-*O*-methyl ether; (**8**) maeruaoside; (**9**) stachydrine; (**10**) 3-hydroxystachydrine; (**11**) 3-hydroxy-1,1-dimethylpyrrolidinium; and (**12**) phloroglucinol-1-*O*-β-glucopyranoside.

The initial screening of seven accessions of *M*. *siamensis* leaf extracts using HPLC-UV-DAD indicated a uniform distribution of specialized metabolites across the collected samples. Based on these preliminary analyses, samples were pooled, ground, and extracted. Chromatographic separation, together with subsequent structure elucidation using 1D- and 2D-NMR experiments combined with mass spectrometry, allowed for the identification of twelve compounds, of which two (**5**, **8**) had not yet been described, from the hydrophilic fraction of the crude methanolic leaf extract (Figure 4). The majority of the known compounds comprised either simple phenolics (**2**–**4**, **12**) or the l-proline-derived compounds stachydrine (**9**), 3-hydroxystachydrine (**10**), and 3-hydroxy-1,1-dimethylpyrrolidinium (**11**) [21]. Because no NMR data for these betaine derivatives were available in literature, a complete resonance assignment was performed. The NMR chemical shifts, including the ^15^N chemical shifts obtained through the ^1^H,^15^N-HMBC experiments, are listed in the Supporting Information (Tables S1 and S2). In addition, the megastigmane blumenol A (**6**) (=vomifoliol; [22]), most likely a degradation product of carotenoids [23], and the flavonol glycoside tamarixetin-3-*O*-β-sophoroside-7-*O*-methyl ether (**7**; [24]) have been identified. The NMR data for this flavonol derivative, measured in MeOD, are also reported in the Supporting Information (Tables S3 and S4).

### 2.4. Structure Elucidation of the Novel Compounds ***5*** and ***8***

Compound **5** showed a peak in the negative mode of the ESI mass spectrum at *m*/*z* = 266.1039 [M-H]^−^ (calcd 266.1029), and in positive mode peaks at *m*/*z* = 290.0996 [M+Na]^+^ (calcd 290.1005), and also at *m*/*z* = 312.0817 [M-H+2Na]^+^ (calcd 312.0842). The disodium adduct was highly characteristic for acids, due to the formation of a Na^+^ adduct of the carboxylic acid sodium salt. All three detected mass peaks corresponded to the molecular formula C_13_H_17_NO_5_. The ^1^H NMR spectrum revealed the typical pattern of a *para*-substituted benzene with the 2H doublets at *δ*_H_ 6.70 and 7.02, respectively. In addition, two triplets with an integral of 2H each at *δ*_H_ 3.32 and 2.68 indicated the presence of a CH_2_-CH_2_ group whereas a second aliphatic spin system could be assigned to a CH_2_-CH_2_-CH-O moiety based on detailed analyses of ^1^H and ^13^C chemical shifts in combination with COSY and HSQC spectra. In addition to the above-mentioned spin systems, the ^13^C NMR spectrum showed two additional resonances of carboxyl carbons at *δ*_C_ 176.1 and 180.7. Finally, the connectivity of the previously identified subunits was obtained via analysis of the HMBC spectrum. A long-range cross-peak from the aromatic protons at *δ*_H_ 7.02 (H3, H5) to a carbon at *δ*_C_ 35.7 (C7) proved the direct attachment of the CH_2_-CH_2_ group to the benzene ring in a phenethylamine moiety. Cross-peaks from H8, H10, and H11 to the carbonyl carbon at *δ*_C_ 176.1 connected the two aliphatic spin systems via an amide bond, whereas a cross-peak from H11 and H12 to the remaining carbonyl signal at *δ*_C_ 180.7 established this carbon to be a terminal carboxylic acid group. All the NMR and mass data of compound **5** were in agreement with the structure shown in Figure 4, which is, to the best of our knowledge, a new phenethylamide derivative. 

The molecular formula of compound **8** was determined to be C_40_H_44_O_21_ based on the ESI [M-H]^−^ mass peak at *m*/*z* = 859.2310 in negative mode (calcd 859.2295), and a [M+Na]^+^ peak at *m*/*z* = 883.2266 in the positive mode (calcd 883.2271). The ^1^H NMR spectrum showed the characteristic *meta*-coupling signals of a 5,7-dioxygenated A-ring of flavonoids at *δ*_H_ 6.18 and 6.15, whereas the B-ring protons revealed a coupling pattern of 3,4-dioxygenation. In the ^1^H NMR spectrum, two anomeric protons at *δ*_H_ 5.26 and 4.73 were detected, indicating the presence of two monosaccharide moieties that were established as β-glucopyranose units with the help of detailed analyses of their chemical shifts and coupling constants. A C,H long-range cross-peak in the HMBC spectrum from the low-field anomeric proton (glc_1_-H1) to C3 at *δ*_C_ 135.6 indicated glycosidation at position *O*-3 of the flavonoid molecule, whereas a long-range cross-peak from the high-field anomeric proton (glc_2_-H1) to C2 of glc_1_ proved the presence of an β-1,2-linked diglucoside, namely 2-*O*-β-d-glucopyranosyl-d-glucopyranoside (sophoroside), itself β-linked to the aglycon. Two methoxyl groups with resonances at *δ*_H_ 3.83 and 3.95 were assigned to positions C7 and C4′ based on NOESY cross-peaks of 7-OMe to H6 and H8 and 4′-OMe to H5′ resulting in the tamarixetin-3-*O*-β-sophoroside-7-*O*-methylether skeleton. Remaining signals in the ^1^H NMR spectrum were two identical methoxyl groups at *δ*_H_ 3.73, two identical aromatic singlet protons at *δ*_H_ 6.43, and signals of a *trans*-configured double bond at *δ*_H_ 7.24 and 5.97, respectively. These signals correspond to a sinapoyl moiety, which is bound as an ester to C6 of glucopyranose-2, as shown by a long-range cross-peak in the HMBC spectrum from glc_2_-H6 to the sinapoyl carboxyl C9″ at *δ*_C_ 168.4. Therefore, based on the mass and NMR data, the chemical structure of compound **8** was determined, as shown in Figure 4. It was found to be a new, not yet described, specialized metabolite. The NMR and mass spectra of compounds **5** and **8** are given in the supplement (Figures S1–S8 for **5** and Figures S9–S20 for compound **8**).

Both tamarixetin derivatives are easily recognized by their characteristic UV spectra in the HPLC-UV-DAD profiles of the crude extract. A comparison of the UV spectra indicates the presence of several other derivatives in much smaller amounts. These small quantities prevented isolation and identification but suggested enhanced structural diversification. The novel tamarixetin glycoside (**8**) is a modular construction of specialized metabolites owing to the sinapinic acid attached to C-6 of glucopyranose-2. The attachment of cinnamic acid and/or closely related compounds, such as caffeic, ferulic, or sinapic acid derivatives, allows wider structural diversification and may contribute to plant fitness.

Azelaic acid (**1**) has previously been found in cereals and *Arabidopsis thaliana* (L.) Heynh. (Brassicaceae). This compound is most likely part of the systematic immunodefense of *A. thaliana* [25] because, after bacterial infections, the *in vivo* concentration of **1** increased and promoted the formation of salicylic acid, a defense chemical against bacteria. A similar role for this compound in woody *Maerua* species is conceivable. Compound **5** could be a degradation product of a phenylethyl amide called (*E*,*E*)-*N*,*N*-dityramin-4,4-dihydroxy-3,5-dimethoxy-β,3-bicinnamamide [26], which has previously been isolated from the leaves of *Aptenia cordifolia* (L.f.) Schwantes (Aizoaceae) [27].

The structural diversity of the identified simple phenolics **2**–**4** and **12** points most likely to be degradation metabolites of the glucosinolate epiglucobarbarin [28], although biosynthesis of such compounds apart from the pathway leading to aromatic glucosinolates cannot be excluded. The compounds reported in previous studies [8,9], together with the close relationship between Capparaceae and Brassicaceae [29], indicate the possible presence of aromatic glucosinolates in the investigated plant species, although they have not yet been confirmed in fresh leaves of *Maerua* species. In this regard, Mithen et al. [10] analyzed herbarium vouchers of *M*. *angolensis*, *M*. *crassiflora*, and *M*. *triphylla* and many other species from the order Brassicales by GC-MS and reported methyl glucosinolate and indol-3-yl methyl glucosinolate as major glucosinolates in the tissues of these three *Maerua* species. The latter compound is most likely the precursor of the indole nitrile glycosides cappariloside A and B [28], which have been reported in the leaves of *M*. *siamensis* [9]. Because the drying process of plant tissue is directly linked to the loss of cell integrity, the isolation of such degradation products is very likely in our case [30]. 

The presence of l-proline-derived betaines (**9**–**11**) [21] in the NMR data (Tables S1 and S2; Figure S21) of the leaf tissue is of special interest because of their close relationship to primary metabolism. The amino acid l-proline content in plant tissues is elevated during osmotic stress [31]. A similar effect was observed in *M*. *oblongifolia* seedlings [32]. Exposure of these seedlings to higher salt concentrations increased the concentration of l-proline and other osmolytes, such as soluble sugars in the leaves. The structural similarity of l-proline and the identified l-proline-derived betaines (**9**–**11**) suggests an analogous function in *M*. *siamensis*. Since *M*. *siamensis* occurs in hot and dry areas on limestone mountains in northeastern Thailand and along the sea shore [12], these compounds may help the plants to mitigate drought stress caused by the lack of precipitation and by the elevated salinity along the sea shores, which are the major causes of osmotic stress in plant cells.

### 2.5. Bioactivities

#### 2.5.1. Antioxidative Assay

The DPPH assay of the crude methanolic extract exhibited an EC_50_ value of 106 µg mL^−1^ (ascorbic acid 4.6 µg mL^−1^), indicating some potential for radical scavenging activities. Yonbawi et al. [33] reported an EC_50_ of 448 µg mL^−1^ for aerial parts of *M. crassifolia*. Because the plant material investigated in the cited work is unclear, a direct comparison of these values is impossible. Compound **10** did not show any decolorization of DPPH at a concentration of 2 mg mL^−1^, indicating inactivity. Both flavonoids lack the structural features required for powerful antioxidative properties [34,35]. 

#### 2.5.2. Insect Feeding Assay Against *S. littoralis*

The assayed crude methanolic extract resulted in growth inhibition of 38% for a testing concentration of 0.9 mg g^−1^, 35% (1.8 mg g^−1^), 29% (2.7 mg g^−1^), and 25% at 3.6 mg g^−1^. In comparison, the positive control nicotine exhibited a growth reduction of 46% at 0.9, 52% (1.8), 72% (2.7), and 100% at 3.6 mg g^−1^. These results do not provide clear evidence for the presence of strong antifeedant active compounds in the investigated plant extracts.

#### 2.5.3. Nematocidal Assay

The initially tested compounds did not increase *Panagrellus redivius* lethality. The positive control, nicotine, exhibited a mortality rate of 89.3% at a concentration of 2.1 mg mL^−1^, and 44.4% at 0.6 mg mL^−1^. These results suggest that the bioactivity of the isolated compounds, especially the l-proline-derived betaines, lies in the prevention of negative effects in cells caused by drought stress rather than in the defense against potential herbivores.

## 3. Materials and Methods

### 3.1. Study Site and Sampling

Fresh plant materials of *M. siamensis* were collected from known localities in four provinces in Thailand in 2020 to 2022 (Table 1). The plants were selected non-systematically due to their heterogeneous distribution throughout the landscape. The species was not known to exhibit clonal growth, as it predominantly reproduces sexually and disperses via seeds. The majority of plants possess hermaphroditic flowers, but rarely produce fruits, indicating the potential requirement for cross-pollination by animal vectors [36]. To ensure the sampling of distinct individuals, the sampled plants were all at least 10 m apart from each other. At least one branch from each tree was collected to make a voucher specimen and provide fresh material for DNA extraction.

The collected specimens were stored in the herbarium of the Department of Botany, Kasetsart University, Bangkok, Thailand. In this study, we defined the populations by the administrative provinces of Thailand, as they were geographically isolated areas at least 100 km apart (Table 1). The collected plant specimens were identified using the key and descriptions in the Flora of Thailand [11].

### 3.2. Phylogenetic and Genetic Diversity Analysis

#### 3.2.1. DNA Extraction, Amplification, and Sequencing

Genomic DNA was extracted from fresh or silica-dried plant material using a Nucleospin^®^ Plant II Kit (Macherey-Nagel GmbH & Co. KG, Düren, Germany), following the manufacturer’s protocol. Two plastid regions, *matK* and *rbcL*, were amplified from genomic DNA extracts. The primers for amplification and sequencing of *the matK* region were as follows: forward primer: 5′ ACCCAGTCCAT CTGGAAATCTTGGTTC 3′ and reverse primer: 5′ CGTACAGTACTTTTGTGTTTACGAG 3′. The PCR protocol for the amplification of *matK* was set as follows: 94 °C for 5 min, 35 cycles (94 °C for 30 s, 52 °C for 20 s, and 72 °C for 1 min), and a final extension at 72 °C for 10 min. Amplification of the *rbcL* region used the forward primer (rbcLa-F): 5′ ATGTCA CCACAAACAGAGACTAAAGC 3′ and the reverse primer (rbcLa-R): 5′ GTAA AATCAAGTCCACCRCG 3′. The PCR protocol was as follows: 94 °C for 4 min, 35 cycles (94 °C for 30 s, 55 °C for 1 min, and 72 °C for 1 min), and a final extension at 72 °C for 10 min. PCR products were checked on a 1% ethidium bromide-free agarose gel (GenedireX^®^ Agarose Tablets, Taoyuan, Taiwan) under an LED transilluminator and cleaned with ExoSAP-IT PCR Product Cleanup (Applied Biosystems, Santa Clara, CA, USA), following the manufacturer’s instructions. The purified PCR products were bidirectionally sequenced using the same primers as those used for amplification. Sanger Sequencing was performed at Macrogen, Inc., Seoul, Republic of Korea.

#### 3.2.2. Phylogenetic Analyses and Test of Population Genetic Structures

To assess the phylogenetic placement of *M. siamensis* representatives, *matK* and *rbc*L sequences were concatenated using MEGA-X [33]. We then constructed the maximum likelihood (ML) phylogeny with RAxML v8.2.12 [37] for 1000 bootstrap pseudo-replicates. The Bayesian inference (BI) phylogeny was also reconstructed with MrBayes v3.2.7a [38], and the Markov Chain Monte Carlo chains were set for 2,000,000 generations, sampled trees every 1000 generations, four chains, temperature of 0.2, and burn-in of 25%. Both analyses were performed on the High-performance Computing Facility of Faculty of Science, Kasetsart University (SciKU HPC).

For each population, we estimated population genetics statistics, including the Shannon-Wiener diversity index, expected heterozygosity, nucleotide diversity, and Tajima’s D, using commands from R-packages APE [39], PEGAS [40], and POPPR [41] in the R environment v 3.6.1 [42]. These genetic diversity measures calculated the variation of the obtained sequences within each defined population. Subsequently, we called all single nucleotide polymorphism sites from a concatenated alignment for the estimation of individual admixture coefficients using sparse non-negative matrix factorization (SNMF) algorithms [43] using the LEA package [44] under the scenario of K = 1 to 6 ancestral populations. The run with the lowest cross-entropy value was considered the most optimal run for visualizing haplotype frequencies within *M. siamensis* populations.

### 3.3. Phytochemical Approach

#### 3.3.1. Chromatographic Procedures

HPLC analyses were performed using an Agilent 1100 series (Agilent Technologies, Waldbronn, Germany) instrument with UV-diode array detection. To analyze the ethyl acetate fraction and monitor all the separation steps from this fraction, we used a Hypersil BDS-C18 (250 × 4.6 mm, 5 μm particle size; Agilent, St. Clara, CA, USA) column at a flow rate of 1.0 mL min^−1^ and an injection volume of 10 µL. An aqueous solution containing 10 mM ammonium acetate (A) and methanol (MeOH) (B) was used as the eluent, and the following gradient was used: from 20 to 90% B in A within 15 min, within 0.1 min to 99% B in A, and 99% B was kept for 9.6 min. The wavelength of detection was set at 230 nm. For analyses of the water phase and monitoring all the separations steps from this fraction, we used a Hypersil BDS-C18 column (100 × 4.0 mm, 3 μm particle size; Agilent, St. Clara, CA, USA) and the eluents were an aqueous buffer (15 mM H_3_PO_4_ and 1.5 mM Bu_4_NOH) (A) and methanol (B) with a flow rate of 0.5 mL min^−1^. The gradient was set as follows: from 15 to 70% B in A within 10 min, within 1 min to 72% B in A, and from 72% to 98% B in A in 0.1 min. Finally, 98% B in A was maintained for 6 min. The injection volume was 5 µL. The wavelength of detection was set at 230 nm.

TLC was performed on precoated silica gel 60 F_254_ plates with 0.20 mm thickness for analytical purposes and 0.5 mm thickness (Merck KGaA, Darmstadt, Germany) for preparative purposes. The plates were developed either in CHCl_3_/MeOH 98:2 or 70:30 (*v*/*v*). Sephadex LH-20 (GE HealthCare, IL, USA) with methanol as the eluent was used for size exclusion chromatography (SEC). Other stationary phases for column chromatography (CC) were silica gel 60 (Merck KGaA, Darmstadt, Germany), either with 0.2–0.5 mm or 40–63 μm particle size, and aluminum oxide (neutral) (Merck KGaA, Darmstadt, Germany). All the separation steps were monitored using TLC combined with UV detection and sprayed with anisaldehyde reagent, Dragendorff’s reagent, and HPLC analysis.

#### 3.3.2. Plant Material for Phytochemical Investigations

*M. siamensis* leaves were collected from Nakhon Ratchasima, Thailand (14.6850782° N, 101.416047° E) for phytochemical investigations. The respective herbarium vouchers of the investigated plant material were deposited at the Bangkok Forest Herbarium (BKF) with the number BKF No. 084907 (N. Wongthet, K. Limkittikul & P. Tiwutanon No. 07).

#### 3.3.3. Nuclear Magnetic Resonance (NMR) and Mass Spectrometry (MS)

For NMR spectroscopic measurements, each sample was dissolved in CD_3_OD (~3.0 mg in 0.7 mL) and transferred into 5 mm high precision NMR sample tubes. The spectra were measured on a Bruker Avance III 600 at 600.25 MHz (^1^H) or 150.94 MHz (^13^C) or on a Bruker Avance III 700 at 700.40 MHz (^1^H) or 176.13 MHz (^13^C), respectively, and processed using the Topspin 3.5 software. 1D spectra were recorded by acquisition of 32k data points and after zero filling to 64k data points, Fourier transformation spectra were performed with a range of 7200 Hz (^1^H) and 32,000 Hz (^13^C). To determine the 2D COSY, TOCSY, NOESY, HSQC, and HMBC spectra, 128 experiments with 2048 data points each were recorded and Fourier-transformed into 2D-spectra with a range of 6000 Hz and 32,000 Hz for ^1^H and ^13^C NMR, respectively. The measurement temperature was 298 K ± 0.05 K. ^1^H,^15^N HMBC spectra were acquired on an Avance II 400 at 400.13 MHz for ^1^H and 40.55 MHz for ^15^N with the N-H long-range coupling constant set to 5 Hz. Incompletely deuterated methanol (CHD_2_OD) was used as the internal standard for ^1^H (*δ*_H_ 3.31) and CD_3_OD was used for ^13^C (*δ*_C_ 49.0) measurements.

Mass spectra were recorded on a high-resolution time-of-flight (HR-TOF) mass spectrometer maXis, (Bruker Daltonics GmbH & Co. KG, Bremen, Germany) by direct infusion electrospray ionization (ESI) in positive ionization mode (mass accuracy ± 5 ppm) as well as in negative mode (mass accuracy ± 10 ppm). HR-TOF MS measurements were performed within a selected mass range of *m*/*z* 100–2500. ESI used a capillary voltage of 4 kV to maintain a (capillary) current between 30 and 50 nA. The nitrogen temperature was maintained at 180 °C using a flow rate of 4.0 L min^−1^ and N_2_ nebulizer gas pressure of 300 hPa.

#### 3.3.4. Antioxidative Assay

The experiment was performed according to the method described by Sudžuković et al. [45]. A dilution series starting from 10 mg mL^−1^ was prepared from crude extracts obtained from the leaves. The leaf extract was tested in the concentration range of 1.0 mg mL^−1^ and 0.49 µg mL^−1^. After measuring the blank, 50 µL of freshly prepared methanolic 2,2-diphenyl-1-picrylhydrazyl (DPPH) solution (0.4 mM) was added to each well. The absorbance of the microwell plates was measured at 517 nm 30 min after adding the stable radical solution using an Infinite M Nano plate reader (Tecan Trading AG, Männedorf, Switzerland). Ascorbic acid was used as the positive control.

#### 3.3.5. Insect Feeding Assay

The insect assay was performed in triplicate with slight modifications, according to Kornpointner et al. [46]. Briefly, 367 mg of freeze-dried food powder, containing ground white beans, yeast, ascorbic acid, and ethyl 4-hydroxybenzoate as preservative, was spiked with 0.9, 1.8, 2.7, and 3.6 mg g^−1^ of the of the crude extracts. After evaporating the solvent (MeOH, 16 h), an aqueous solution containing vitamins and chloramphenicol was added. Ten freshly hatched larvae of the cotton leafworm *Spodoptera littoralis* were placed on each food pellet in separate Petri dishes and kept in an incubator at 226 °C and 90% humidity in the dark for 96 h. The mass of the surviving larvae was evaluated, and the average percentage of weight gained was calculated. Nicotine was used as a positive control.

#### 3.3.6. Nematocidal Assay

Both identified flavonol glycosides **7**, **8**, together with **10** and l-proline, were bioassayed against *Panagrellus redivivus*. Nicotine served as a positive control and dH_2_O, respectively, and a 5% (*v*/*v*) DMSO solution served as a negative control. Accurately weighed compounds were dissolved in dH_2_O to reach a concentration of 2.0 mg mL^−1^. Nicotine was dissolved in DMSO and water was added to reach a concentration of 5% DMSO in dH_2_O to reach a conc. of 3 mg mL^−1^. In a flat-bottom microwell plate, 10 µL of nematode suspension containing 10–20 nematodes was transferred into each well; the plates were initially photographed using a binocular, and the number of nematodes were counted. Subsequently, 40 µL of each stock solution was added to reach a concentration of 80 µg of isolated compounds per well. Nicotine was finally tested at 2.1 and 0.6 mg mL^−1^. The plates were then incubated at room temperature for 24 h. Subsequently, the plates were photographed again and the straight-shaped nematodes (dead nematodes) were counted from the obtained photographs. Finally, the percentage of survivors was calculated. The assay was performed in triplicate.

#### 3.3.7. HPLC Screening, Extraction, and Isolation

Initial screening by HPLC-UV-DAD was performed according to a published protocol [47], with slight modifications. In brief, 20 ± 1 mg of ground leaves from seven accessions was extracted with 500 µL MeOH in Eppendorf tubes by sonication for 20 min. After centrifugation at 12,500 rpm for 15 min, the supernatant was transferred to Eppendorf tubes and the extraction step was repeated. The pooled extracts were dried overnight, and the amount of extract was determined and re-dissolved in MeOH at a concentration of 2 mg mL^−1^ prior to HPLC measurement. To analyze the hydrophilic phase, the obtained extracts were transferred back to Eppendorf tubes, the solvent was evaporated, and 500 µL of dH_2_O was added. Subsequently, 500 µL of CHCl_3_ was added, the tubes were closed, shaken, centrifuged at 12,000 rpm for 15 min, and the supernatant was subjected to HPLC analysis. Based on these analyses, the collected air-dried leaves were pooled (327 g), ground, soaked in methanol, and filtered after 3 days. This step was repeated twice, and the solvent was evaporated from the combined extracts using a rotary evaporator. This yielded a total of 18.2 g of brownish residue. This residue was suspended in water, and the subsequent extraction with chloroform afforded 6.7 g of lipophilic phase, 0.3 g of ethyl acetate phase, and 11.2 g of hydrophilic phase.

#### 3.3.8. Chromatographic Separation of the Ethyl Acetate Phase

The obtained ethyl acetate fraction was chromatographed over silica gel 60 (Column label K01) eluted with 50 mL mixtures of *n*-heptane, ethyl acetate, and MeOH at 100:0:0, 80:20:0, 60:40:0, 50:50:0, 40:60:0, 30:70:0, 20:80:0, 10:90:0, 0:100:0, 0:80:20, and 0:100:0 (*v*/*v*/*v*). Nineteen fractions (30 mL each) were collected. The fraction K01-II_2_ contained 1.6 mg of **4** MK-MS05 and the fractions K01-III_2_ and MK-K01-IV_1_ contained 1.2 mg of **1** MK-MS01 and 2.6 mg of **2**. Compounds **6** (1.2 mg) and **4** were purified by preparative TLC from the combined fractions K01-V_1_ and V_2_ (12.3 mg). Compounds **7** (3.6 mg) and **8** (2.1 mg) were purified by SEC in MeOH from fractions K01-IX_2_ and K01-X_1_, respectively. Compound **5** (8.1 mg) was obtained by SEC and eluted with methanol from the fraction K01-X_2_ (43.1 mg).

#### 3.3.9. Chromatographic Separation of the Water Phase

A portion of the aqueous phase (4.3 g) was adsorbed onto aluminum oxide. This powder was subsequently subjected to column chromatography over aluminum oxide and eluted with CHCl_3_/MeOH mixtures in ratios of 100:0, 90:10, 85:15, 80:20, 70:30, 55:45, 50:50, and 0:100 (*v*/*v*). Twenty-five fractions of 50 mL each were collected. Fractions AL01–04–08 were pooled (0.57 g) and subjected to SEC in MeOH. Fraction 5 contained 15.2 mg of a mixture of **9** and **11** at a ratio of 5:3. Fraction AL01–20 (18.3 mg) was finally purified by SEC. Fractions 1–3 yielded 10.0 mg of **10** (mixture of isomers), and fractions 9–12 afforded 1.6 mg of **11**. The combined fractions AL01–13+14 (134 mg), containing impure **10**, was subjected to SEC in methanol. Fractions 1–8 were pooled, the methanol evaporated, and the remainder was dissolved in dH_2_O. This aqueous solution was extracted with chloroform to remove lipophilic impurities, which yielded 61 mg of **10**. 

The remaining aqueous phase (6.9 g) was adsorbed on 5 g silica gel (0.2–0.5 mm grain size) and this powder was further subjected to CC packed with 30 g silica gel 60 (40–60 µm particle size) suspended in chloroform and eluted with mixtures of chloroform and methanol in the ratios (0:100, 25:75, 50:50, 0:100; 150 mL each). This step furnished 38 fractions between 10 and 20 mL. Fractions 36–38 were pooled and contained compound 3.9 mg of **12**.

### 3.4. Spectroscopic Data of the Novel Compounds

#### 3.4.1. Maeruamide (**5**)

^1^H (MeOD, 700 MHz), *δ* [ppm]: 1.85 (m, 1H, H-11a), 2.02 (m, 1H, H-11b), 2.23 (ddd, *J* = 14.2, 10.2, 5.6, 1H, H-10a), 2.29 (ddd, *J* = 14.2, 10.2, 6.2, 1H, H-10b), 2.68 (t, *J* = 7.4, 2H, H-7), 3.32 (t, *J* = 7.4, 2H, H-8), 3.91 (dd, *J* = 7.0, 4.2, 1H, H-12), 6.70 (d, *J* = 8.6, 2H, H-2, H-6), 7.02 (d, *J* = 8.6, 2H, H-3, H-5).

^13^C (MeOD, 176 MHz), *δ* [ppm]: 32.3 (C-10), 32.4 (C-11), 35.7 (C-7), 42.4 (C-8), 72.7 (C-12), 116.2 (C-2, C-6), 130.7 (C-3, C-5), 131.3 (C-4), 156.9 (C-1), 176.1 (C-9), 180.7 (C-13); λ_max(H2O/MeOH)_ 222, 276 nm.

#### 3.4.2. Maeruaoside (**8**)

^1^H (MeOD, 700 MHz), *δ* [ppm]: 3.11 (ddd, *J* = 9.7, 5.4, 2.1, 1H, Glc_1_-H5), 3.34 (dd, *J* = 9.7, 9.1, 1H, Glc_1_-H4), 3.37 (t, *J* = 9.3, Glc_2_-H4), 3.43 (dd, *J* = 8.8, 7.5, 1H, Glc_2_-H2), 3.47 (m, 1H, Glc_1_-H6a), 3.48 (m, 1H, Glc_2_-H3), 3.54 (t, *J* = 9.1, 1H, Glc_1_-H3), 3.62 (dd, *J* = 11.9, 2.1, 1H, Glc_1_-H6b), 3.67 (dd, *J* = 9.1, 7.8, 1H, Glc_1_-H2), 3.73 (s, 6H, 3″-OMe, 5″-OMe), 3.75 (m, 1H, Glc_2_-H5), 3.83 (s, 3H, 7-OMe), 3.95 (s, 3H, 4′-OMe), 4.42 (dd, *J* = 11.8, 2.8, 1H, Glc_2_-H6a), 4.45 (dd, *J* = 11.8, 6.5, 1H, Glc_2_-H6b), 4.73 (d, *J* = 7.5, 1H, Glc_2_-H1), 5.26 (d, *J* = 7.8, 1H, Glc_1_-H1), 5.97 (d, *J* = 15.8, 1H, H8″), 6.15 (d, *J* = 2.1, 1H, H8), 6.18 (d, *J* = 2.1, 1H, H6), 6.43 (s, 2H, H2″, H6″), 7.02 (d, *J* = 8.5, 1H, H5′), 7.24 (d, *J* = 15.8, 1H, H7″), 7.61 (d, *J* = 2.2, 1H, H2′), 7.74 (dd, *J* = 8.5, 2.2, 1H, H6′).

^13^C (MeOD, 176 MHz), *δ* [ppm]: 56.3 (4′-OMe), 56.42 (7-OMe), 56.45 (3″-OMe, 5″-OMe), 62.3 (Glc_2_-C6), 64.7 (Glc_1_-C6), 71.1 (Glc_1_-C4), 72.1 (Glc_2_-C4), 75.6 (Glc_2_-C5), 76.4 (Glc_2_-C2), 77.6 (Glc_1_-C3), 77.7 (Glc_2_-C3), 78.3 (Glc_1_-C5), 85.2 (Glc_1_-C2), 99.0 (C6), 92.6 (C8), 100.7 (Glc_1_-C1), 105.6 (Glc_2_-C1), 106.1 (C2″, C6″), 106.5 (C4a), 112.1 (C5′), 115.2 (C8″), 117.1 (C2′), 123.5 (C6′), 124.3 (C1′), 126.1 (C1″), 135.6 (C3), 139.2 (C4″), 146.7 (C7″), 147.3 (C3′), 149.0 (C3″, C5″), 151.6 (C-4′), 157.9* (C8a), 158.0* (C2), 162.5 (C5), 166.9 (C7), 168.7 (C9″), 179.9 (C4). *: exchangeable; λ_max(H2O/MeOH)_ 206, 244, 268, 334 nm.

## 4. Conclusions

Phylogenetic analyses with chloroplast markers confirmed the monophyly of *M. siamensis* and taxonomic identities of specimens collected from Thailand. The analysis of population genetics indicated small genetic variation among the sampled populations, which may be attributed to either purifying selection or recent changes in population demographics. To better understand the mechanisms behind the observed minimal genetic variation in *Maerua*, it would be valuable to gather more data on their reproductive processes. This includes investigating the specific functions of pollinators, such as how bees might encourage geitonogamy and how birds could facilitate xenogamy. Additionally, examining fruiting success rates, seed viability, and potential obstacles to cross-pollination would provide crucial insights, as these aspects are currently not well-understood for *M. siamensis*.

Twelve hydrophilic metabolites belonging to various classes of compounds were identified in the methanolic leaf extracts of *M*. *siamensis* (Capparaceae). Among them, a previously undescribed flavonol glycoside derivative, maeruaoside (**8**), and an amide, maeruamide (**5**), have been described. l-Proline-derived stachydrine (**9**) and its derivatives 3-hydroxy stachydrine (**10**) and 3-hydroxy-1,1-dimethylpyrrolidinium (**11**) were the most abundant nitrogen-containing compounds in the extract. Outstanding bioactivities against various testing organisms were not observed. The crude extract exhibited elevated radical scavenging activity, which may explain the effective use of plant parts in traditional medicine. Investigation of fresh leaves using GC-MS, focusing on glucosinolates, may lead to the identification of such compounds. Nevertheless, further work aimed at the identification of the bioactive principles of this *Maerua* species is required.

## Figures and Tables

**Table 1:** Population statistics from two chloroplast markers of *Maerua siamensis*, including length in base pairs, sample size (N), Shannon-Weiner diversity index (Shannon), expected heterozygosity (H_exp_), nucleotide diversity (π), and Tajima’s D, with associated *p*-values.

Population	N	Shannon	H_exp_	π	Tajima’s D	*p*-Value
Nakhon Ratchasima (NR) *	4	0	0	0	N/A	N/A
Kanchanaburi (KA)	34	3.037	0.175	0.003	−2.210	0.027
Phetchaburi (PB)	11	1.421	0.056	0.005	−0.912	0.362
Bangkok (BKK) *	3	1.099	0.111	0.004	N/A	N/A
Total	52	2.951	0.138	0.002	−2.294	0.022

* Tajima’s D test could not be performed for these populations because of the small populations or identical sequences.

## Data Availability

Data are available upon request.

## Supplementary Materials

The following supporting information can be downloaded at: https://www.mdpi.com/article/10.3390/plants13233359/s1, Table S1. ^1^H chemical shifts of compounds **9**–**11** in CD_3_OD; Table S2. ^13^C and ^15^N chemical shifts of compounds **9**–**11** in CD_3_OD; Table S3. ^1^H chemical shifts of compounds **7** and **8** in CD_3_OD; Table S4. ^13^C chemical shifts of compounds **7** and **8** in CD_3_OD; Figure S1. ^1^H NMR spectrum of **5**; Figure S2. ^1^H NMR spectrum of **5**, expansion; Figure S3. ^13^C NMR spectrum of **5**; Figure S4. COSY NMR spectrum of **5**; Figure S5. edited gs-HSQC NMR spectrum of **5**; Figure S6. gs-HMBC NMR spectrum of **5**; Figure S7. ESI mass spectrum of **5**, negative mode; Figure S8. ESI mass spectrum of **5**, positive mode; Figure S9. ^1^H NMR spectrum of **8**; Figure S10. ^1^H NMR spectrum of **8**, expansion; Figure S11. ^1^H NMR spectrum of **8**, expansion; Figure S12. ^13^C NMR spectrum of **8**; Figure S13. ^13^C NMR spectrum of **8**, expansion; Figure S14. COSY NMR spectrum of **8**; Figure S15. edited gs-HSQC NMR spectrum of **8**; Figure S16. gs-HMBC NMR spectrum of **8**; Figure S17. TOCSY NMR spectrum of **8**, carbohydrate region; Figure S18. gs-NOESY NMR spectrum of **8**; Figure S19. ESI mass spectrum of **8**, negative mode; Figure S20. ESI mass spectrum of **8**, positive mode; Figure S21. ^1^H,^15^N-HMBC spectrum of a mixture of **9**, **10**, and betain.
