# Supplementary material for: Genetic Diversity, Chemical Constituents, and Bioactivities of Maerua siamensis from Thailand
Source: Plants (Basel). 2024 Nov 29;13(23):3359. doi: 10.3390/plants13233359 (PMC11644238; doi:10.3390/plants13233359)
Supplement: Supplementary file 1 [file plants-13-03359-s001.zip › plants-3260319-supplementary.pdf]

Genetic diversity, chemical constituents, and bioactivities of *Maerua siamensis* from Thailand

Supplementary Materials

Natthawadi Wongthet [a], Markus Bacher [b], Mara Krenn [c], Apichat Jai-aree [d], Thomas Rosenau [b], Patsakorn Tiwutanon [a], Nopparat Anantaprayoon [a], Srunya Vajrodaya [a], Lothar Brecker [e], Johann Schinnerl [c,*], Ekaphan Kraichak [a,f,*]

[a] Department of Botany, Faculty of Science, Kasetsart University, Bangkok 10900, Thailand.

[b] Department of Chemistry, Institute of Chemistry of Renewable Resources, University of Natural Resources and Life Sciences (BOKU), Tulln 3430, Austria.

[c] Department of Botany and Biodiversity Research, University of Vienna, Rennweg 14, 1030 Vienna, Austria.

[d] Department of Human and Community Resource Development, Faculty of Education and Development Sciences, Kasetsart University (Kamphaeng Saen Campus), Nakhon Pathom 73140, Thailand.

[e] Department of Organic Chemistry, University of Vienna, Währinger Strasse 38, A-1090 Vienna, Austria.

[f] Biodiversity Center, Kasetsart University, Bangkok 10900, Thailand.

[*] Corresponding authors:

Ekaphan Kraichak (ekaphan.k@ku.th)

Johann Schinnerl (johann.schinnerl@univie.ac.at)

Table of contents

Table S1: ^{1}H chemical shifts of compounds **9**–**11** in CD_3OD

Table S2: ^{13}C and ^{15}N chemical shifts of compounds **9**–**11** in CD_3OD

Table S3: ^{1}H chemical shifts of compounds **7** and **8** in CD_3OD

Table S4: ^{13}C chemical shifts of compounds **7** and **8** in CD_3OD

Figure S1. ^{1}H NMR spectrum of **5**

Figure S2. ^{1}H NMR spectrum of **5**, expansion

Figure S3. ^{13}C NMR spectrum of **5**

Figure S4. COSY NMR spectrum of **5**

Figure S5. edited gs-HSQC NMR spectrum of **5**

Figure S6. gs-HMBC NMR spectrum of **5**

Figure S7. ESI mass spectrum of **5**, negative mode

Figure S8. ESI mass spectrum of **5**, positive mode

Figure S9. ^{1}H NMR spectrum of **8**

Figure S10. ^{1}H NMR spectrum of **8**, expansion

Figure S11. ^{1}H NMR spectrum of **8**, expansion

Figure S12. ^{13}C NMR spectrum of **8**

Figure S13. ^{13}C NMR spectrum of **8**, expansion

Figure S14. COSY NMR spectrum of **8**

Figure S15. edited gs-HSQC NMR spectrum of **8**

Figure S16. gs-HMBC NMR spectrum of **8**

Figure S17. TOCSY NMR spectrum of **8**, carbohydrate region

Figure S18. gs-NOESY NMR spectrum of **8**

Figure S19. ESI mass spectrum of **8**, negative mode

Figure S20. ESI mass spectrum of **8**, positive mode

Figure S21. ^{1}H,^{15}N-HMBC spectrum of a mixture of **9**, **10**, and betain.

Table S1: ^{1}H chemical shifts of compounds **9–11** in CD_3OD, coupling constants are given in Hz.

	9	10<br>isomer-1	10<br>isomer-2	11	betain
2	4.09, dd, *J* = 10.2, 8.9	3.82, d, *J* = 4.5	3.97, d, *J* = 5.5	3.64, dd, *J* = 12.6, 5.4	3.84, s
				3.55, d, *J* = 12.6	-
3	2.50 + 2.31, m	4.73, m	4.76, m	4.67, m	-
4	2.15, m	2.66 + 2.06, m	2.60 + 2.11, m	2.58 + 2.19, m	-
5	3.71 + 3.52, m	3.86 + 3.74, m	3.78 + 3.44, m	3.81 + 3.59, m	-
NCH_3	3.33, s + 3.16, s	3.38 + 3.18, s	3.42 + 3.33, s	3.32, s + 3.20, s	3.28, s

Table S2: ^{13}C and ^{15}N chemical shifts of compounds **9–11** in CD_3OD, coupling constants are given in Hz.

Pos.	9	10a	10b	11	betain
1	170.9	170.0	168.6	-	168.7
2	77.6	85.4	81.3, t, *J* = 2.3	74.6, t, *J* = 3.4	67.2, t, *J* = 4.0
3	26.6	73.7	71.3	70.8	-
4	19.8	32.0	31.2	34.5	-
5	68.0, t, *J* = 3.0	66.3, t, *J* = 2.9	67.0, t, *J* = 3.4	66.7, t, *J* = 3.1	-
NCH_3	52.7, t, *J* = 4.0	54.6, t, *J* = 3.7	53.6, t, *J* = 4.2	55.1, t, *J* = 3.8	53.8, t, *J* = 4.0
	46.3, t, *J* = 3.7	49.2, t, *J* = 3.6	48.8, t, *J* = 3.6	54.5, t, *J* = 4.1	
^{15}N	-311.9	-317.6	-317.6	n.d.	-334.0

Table S3: ^{1}H chemical shifts of compounds **7** and **8** in CD_3OD, coupling constants are given in Hz.

**pos**	**8**	**7**
6	6.18, d, *J* = 2.1	6.61, d, *J* = 2.2
8	6.15, d, *J* = 2.1	6.34, d, *J* = 2.2
2ʻ	7.61, d, *J* = 2.2	7.71, d, *J* = 2.1
5ʻ	7.02, d, *J* = 8.5	7.08, d, *J* = 8.5
6ʻ	7.74, dd, *J* = 8.5, 2.2	7.69, dd, *J* = 8.5, 2.1
Glc_1-1	5.26, d, *J* = 7.8	5.47, d, *J* = 7.7
Glc_1-2	3.67, dd, *J* = 9.1, 7.8	3.76, dd, *J* = 8.9, 7.7
Glc_1-3	3.54, t, *J* = 9.1	3.60, t, *J* = 8.9
Glc_1-4	3.34, dd, *J* = 9.7, 9.1	3.37, m
Glc_1-5	3.11, ddd, *J* = 9.7, 5.4, 2.1	3.21, ddd, *J* = 9.7, 5.5, 2.3
Glc_1-6a	3.62, dd, *J* = 11.9, 2.1	3.51, dd, *J* = 12.0, 5.5
Glc_1-6b	3.47, overlapping m	3.70, m
Glc_2-1	4.73, d, *J* = 7.5	4.77, d, *J* = 7.4
Glc_2-2	3.43, dd, *J* = 8.8, 7.5	3.36, m
Glc_2-3	3.48, m	3.40, m
Glc_2-4	3.37, t, *J* = 9.3	3.40, m
Glc_2-5	3.75, m	3.32, m
Glc_2-6a	4.45, dd, *J* = 11.8, 6.5	3.79, dd, *J* = 11.8, 2.3
Glc_2-6b	4.42, dd, *J* = 11.8, 2.8	3.70, m
2ʻʻ, 6ʻʻ	6.43, s	-
7ʻʻ	7.24, d, *J* = 15.8	-
8ʻʻ	5.97, d, *J* = 15.8	-
7-OMe	3.83, s	3.89, s
4ʻ-OMe	3.95, s	3.95, s
3ʻʻ-, 5ʻʻ-OMe	3.73, s	-

Table S4: ^{13}C chemical shifts of compounds **7** and **8** in CD_3OD, a,b: exchangeable.

pos	7	8	pos	7	8
2	158.6	158.0[a]	Glc$_2$-1	105.1	105.6
3	135.5	135.6	Glc$_2$-2	83.0	76.4
4	179.9	179.9	Glc$_2$-3	77.9	77.7
4a	106.7	106.5	Glc$_2$-4	71.1	72.1
5	162.9	162.5	Glc$_2$-5	78.4	75.6
6	99.1	99.0	Glc$_2$-6	62.4	64.7
7	167.3	166.9	1''	-	126.1
8	93.1	92.6	2'', 6''	-	106.1
8a	158.4	157.9[a]	3'', 5''	-	149.0
1'	124.3	124.3	4''	-	139.2
2'	117.4	117.1	7''	-	146.7
3'	147.2	147.3	8''	-	115.2
4'	151.7	151.6	9''		168.4
5'	112.1	112.1	7-OMe		56.42[b]
6'	123.0	123.5	4'-OMe		56.32[b]
Glc$_1$-1	100.9	100.7	3''-, 5''-OMe		56.45
Glc$_1$-2	83.0	85.2			
Glc$_1$-3	77.9	77.6			
Glc$_1$-4	71.1	71.1			
Glc$_1$-5	78.4	78.3			
Glc$_1$-6	62.4	62.3			

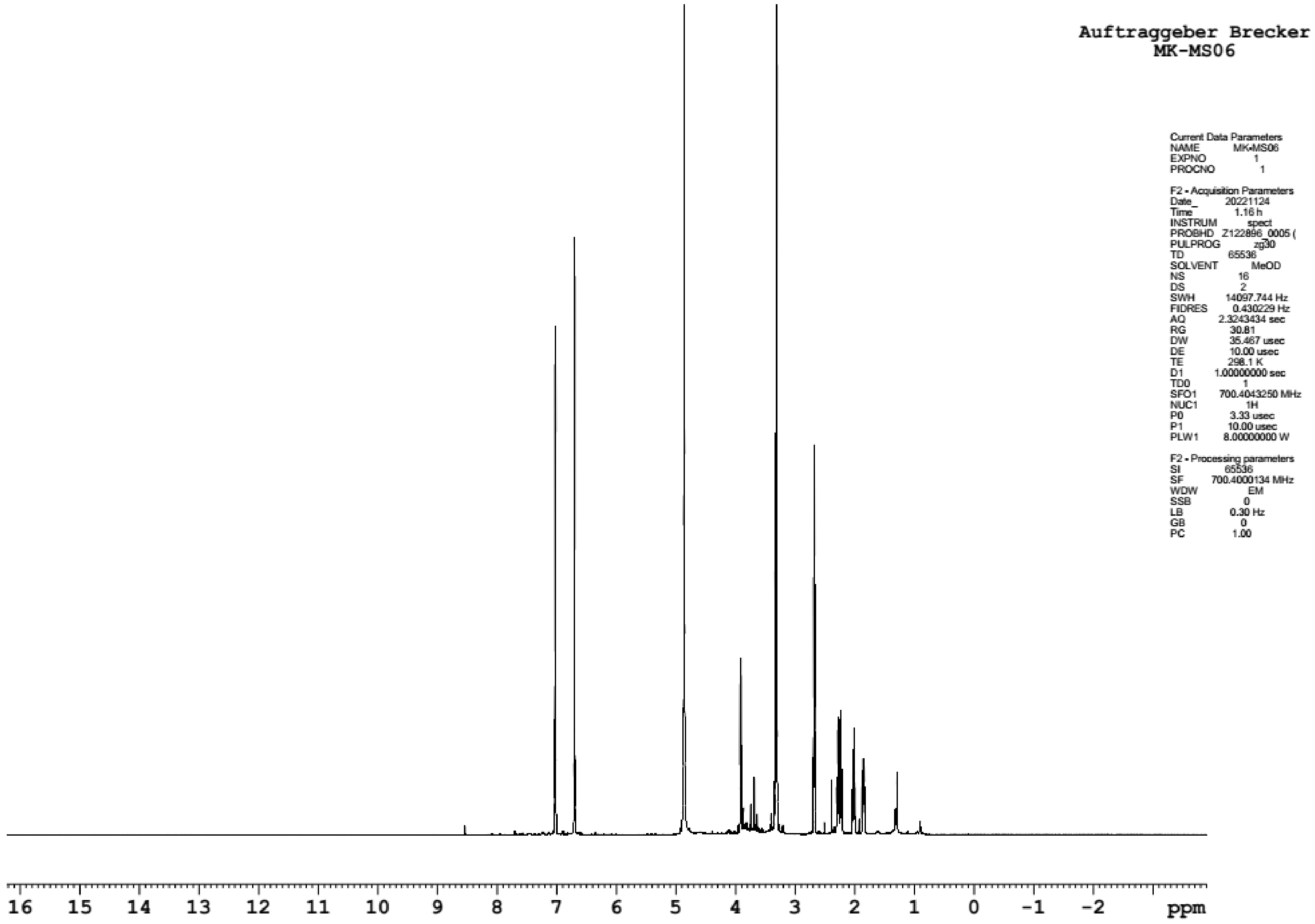

Figure S1. ^{1}H NMR spectrum of **5**.

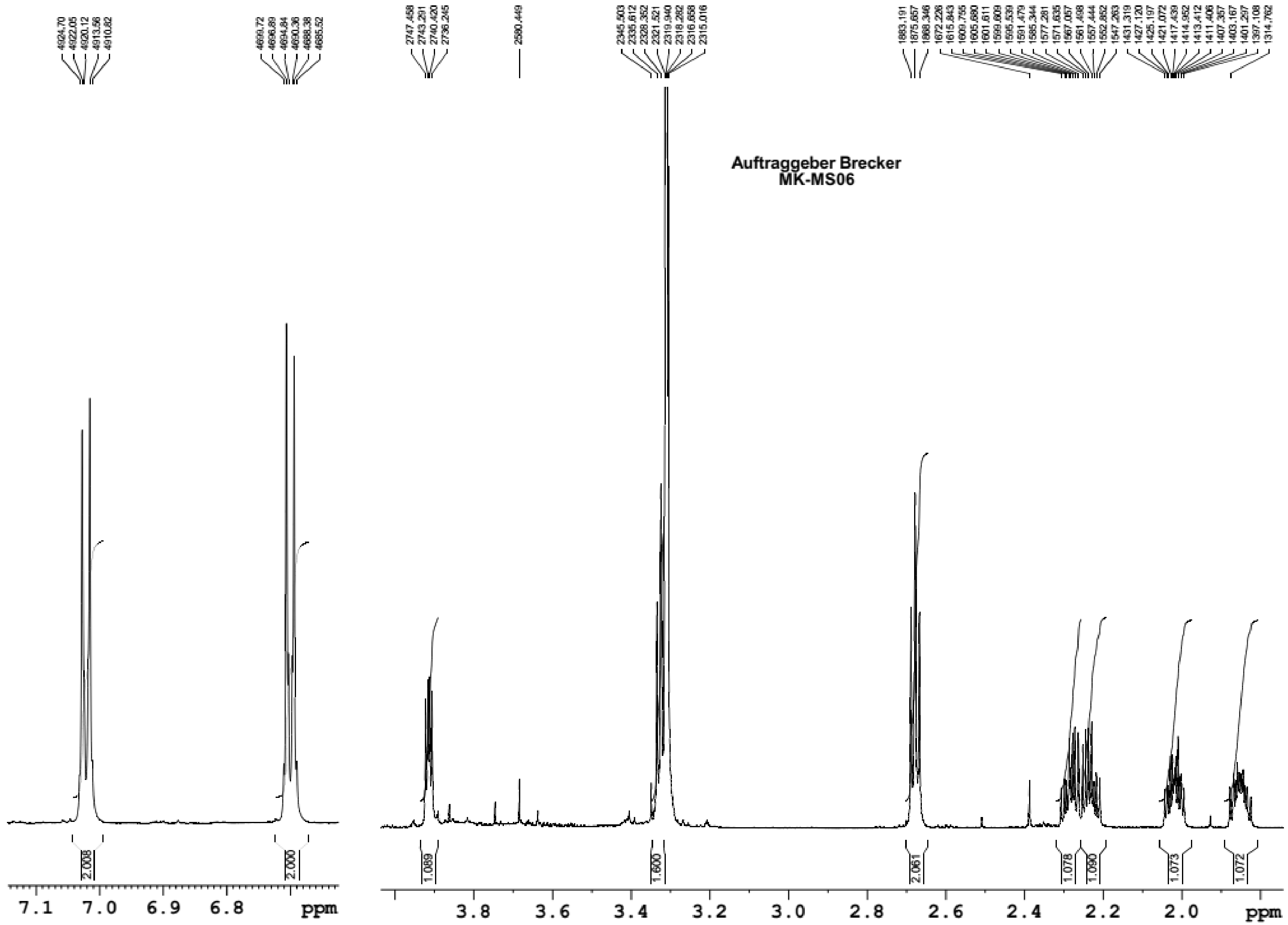

Figure S2. ^{1}H NMR spectrum of **5**, expansion

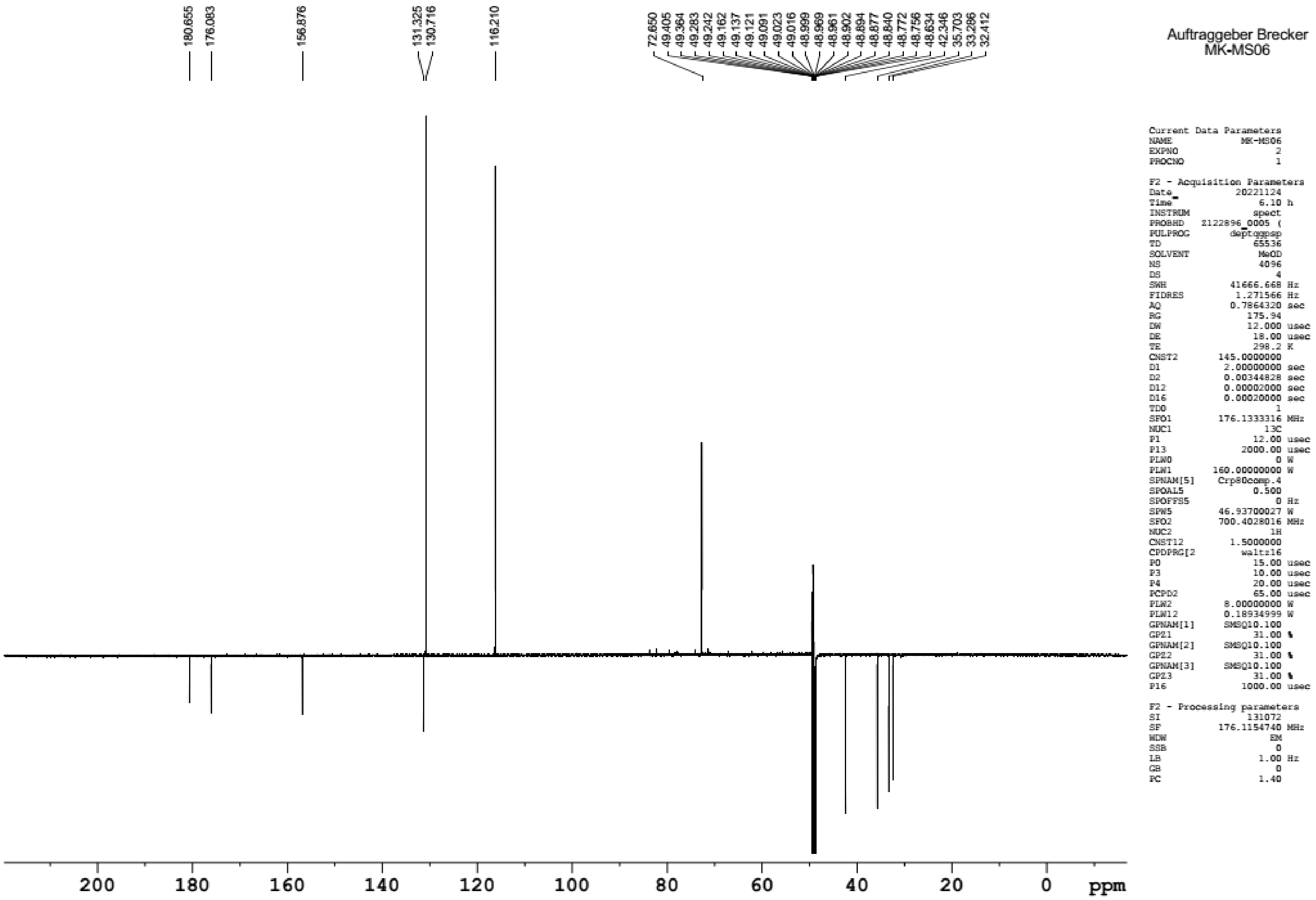

Figure S3. ^{13}C NMR spectrum of **5**.

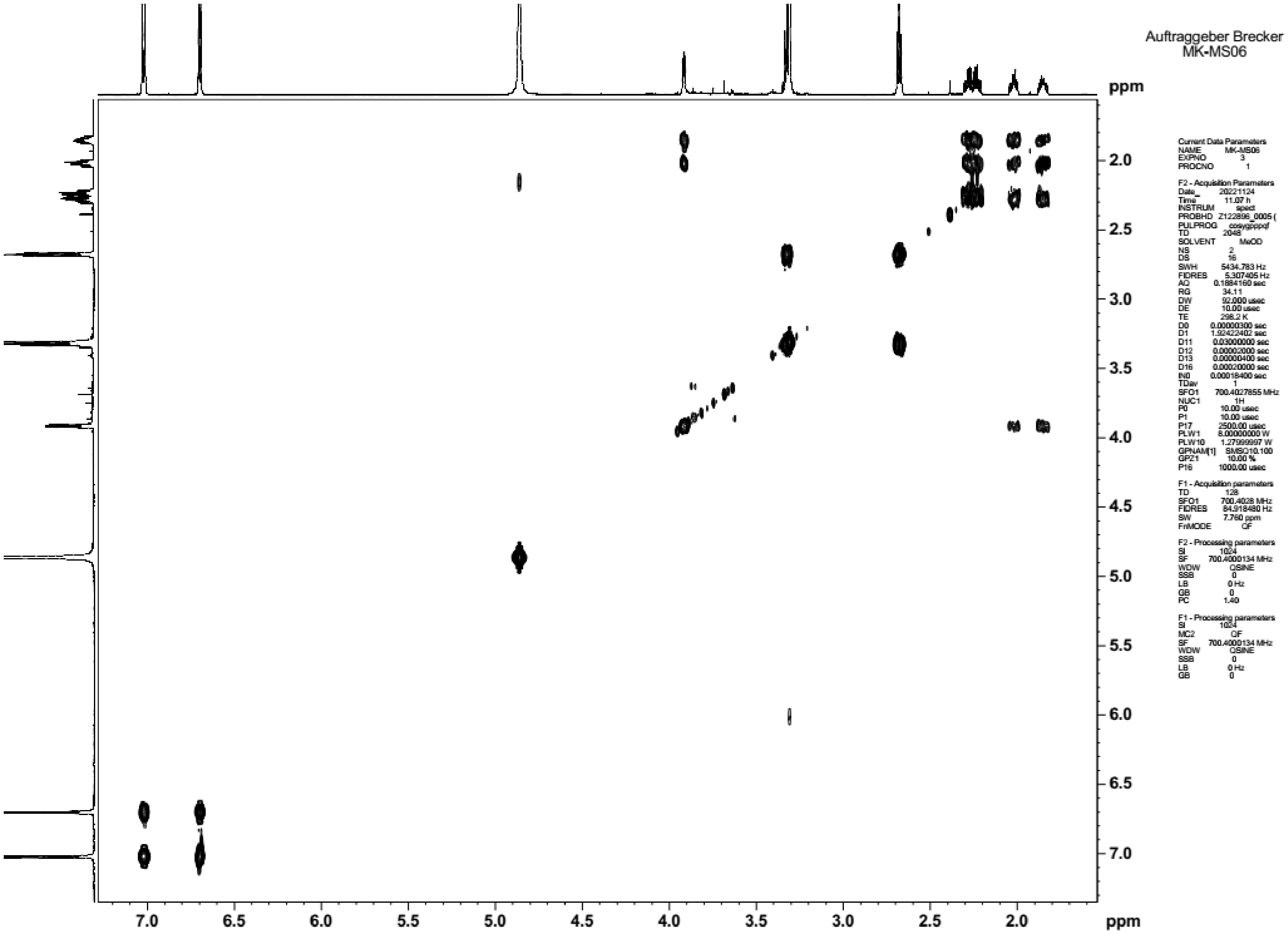

Figure S4. COSY NMR spectrum of **5**.

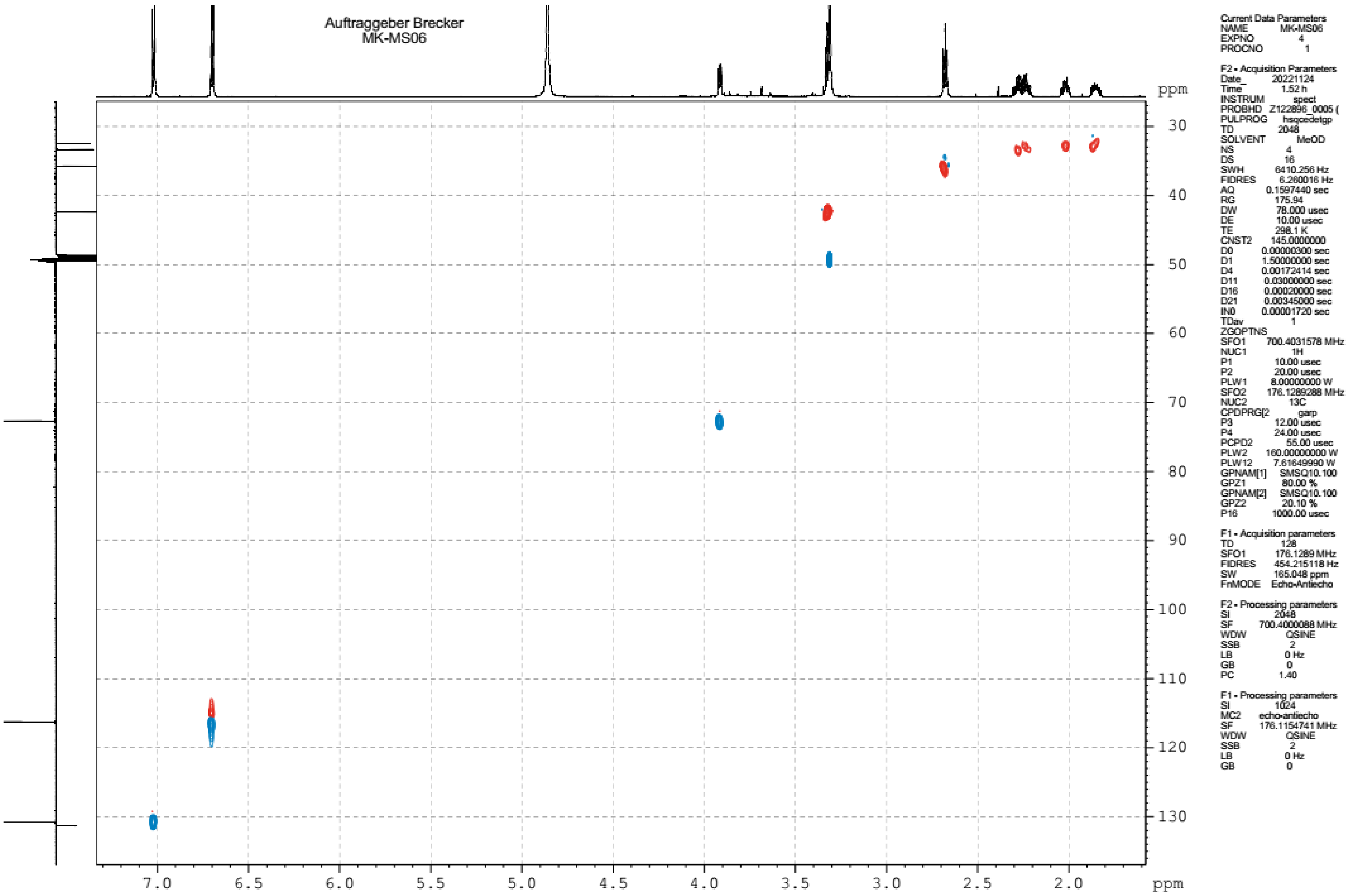

Figure S5. edited gs-HSQC NMR spectrum of **5**.

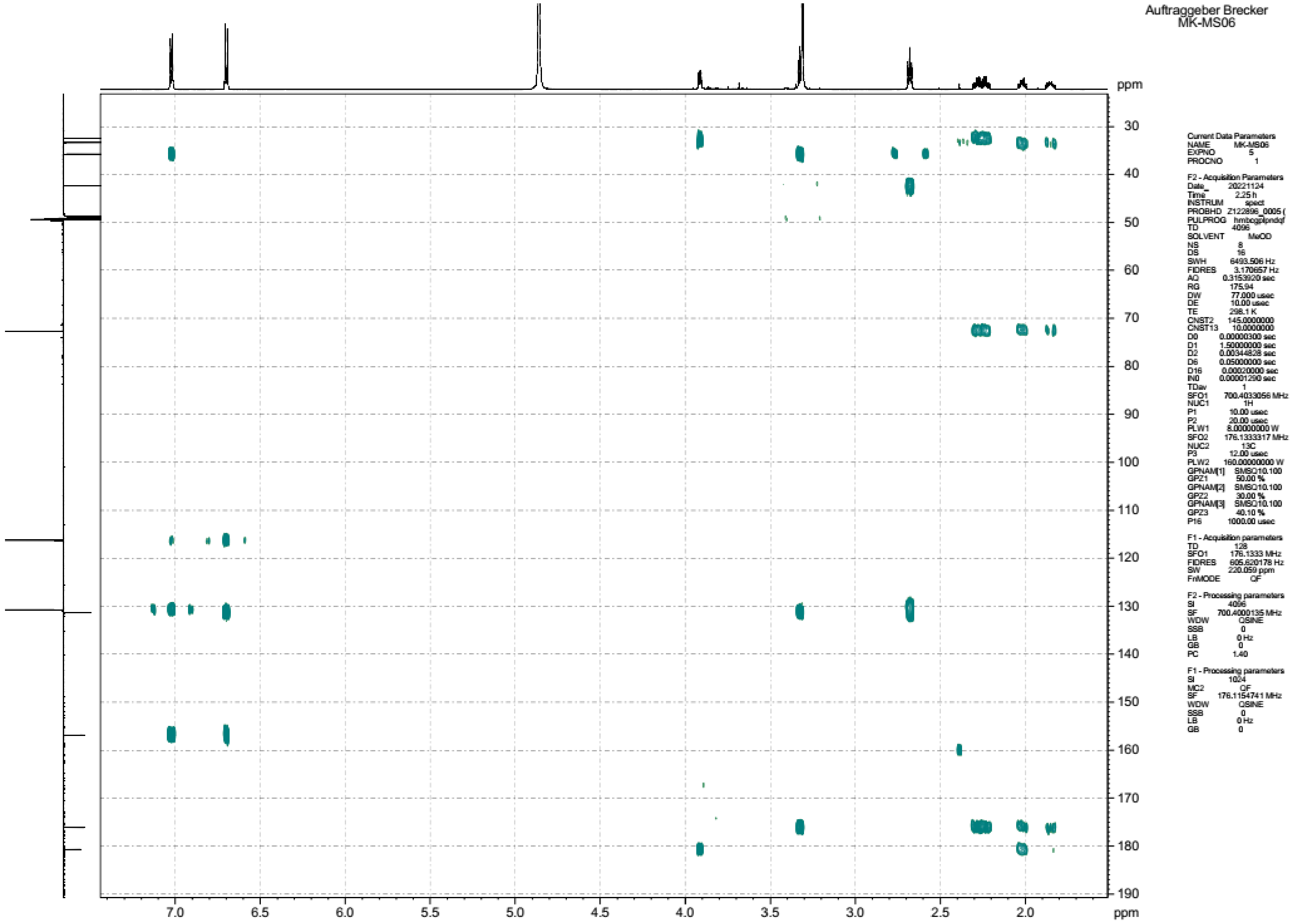

Figure S6. gs-HMBC NMR spectrum of **5**.

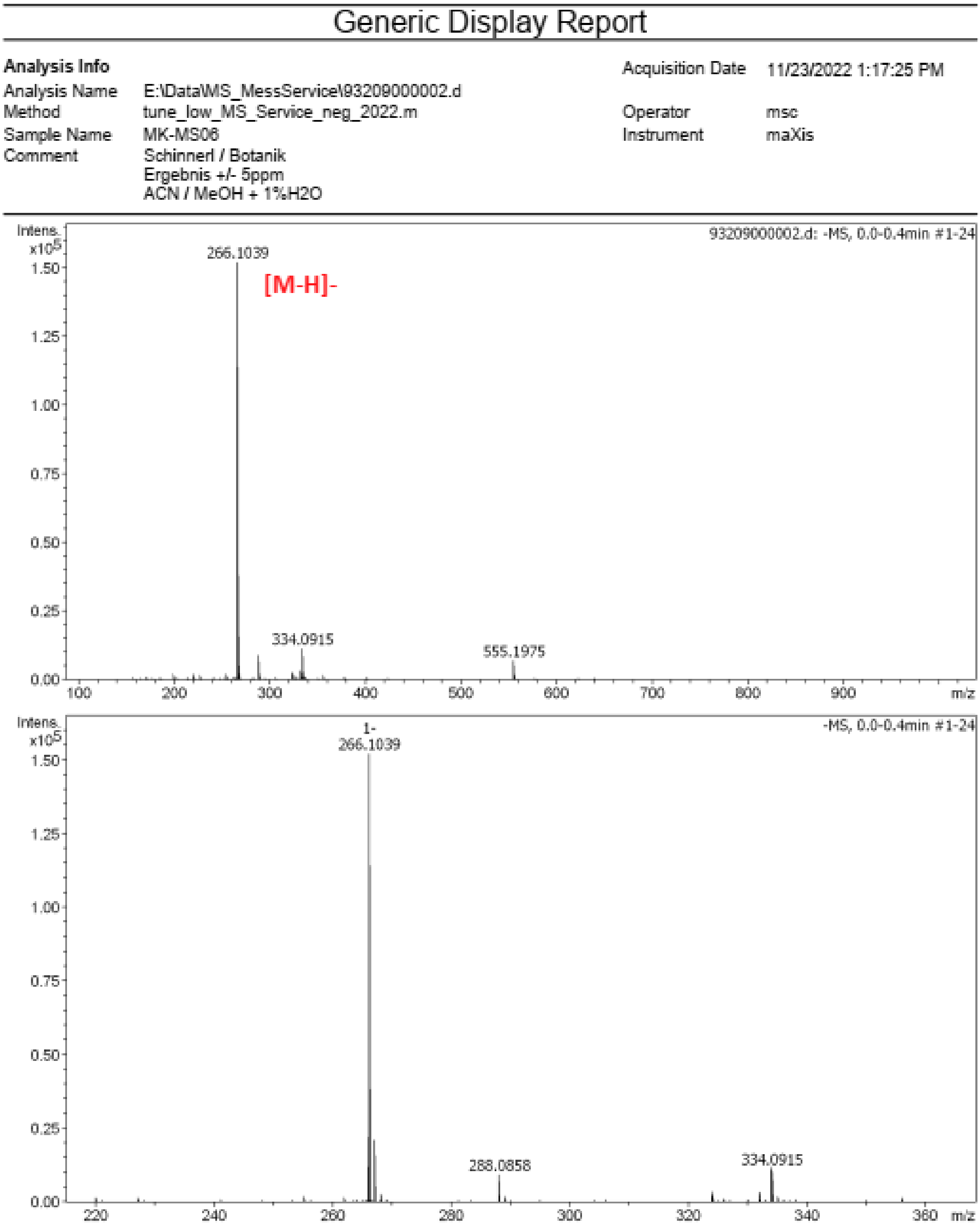

Figure S7. ESI mass spectrum of **5**., negative mode

Generic Display Report

Analysis Info

Analysis Name	E:\Data\MS_MessService\93209000001.d	Acquisition Date	11/22/2022 3:11:46 PM
Method	tune_low_MS_Service_11_22.m	Operator	msc
Sample Name	MK-MS 06	Instrument	maXis
Comment	Schinnerl / Botanik<br>Ergebnis +/- 5ppm<br>ACN / MeOH + 1%H2O		

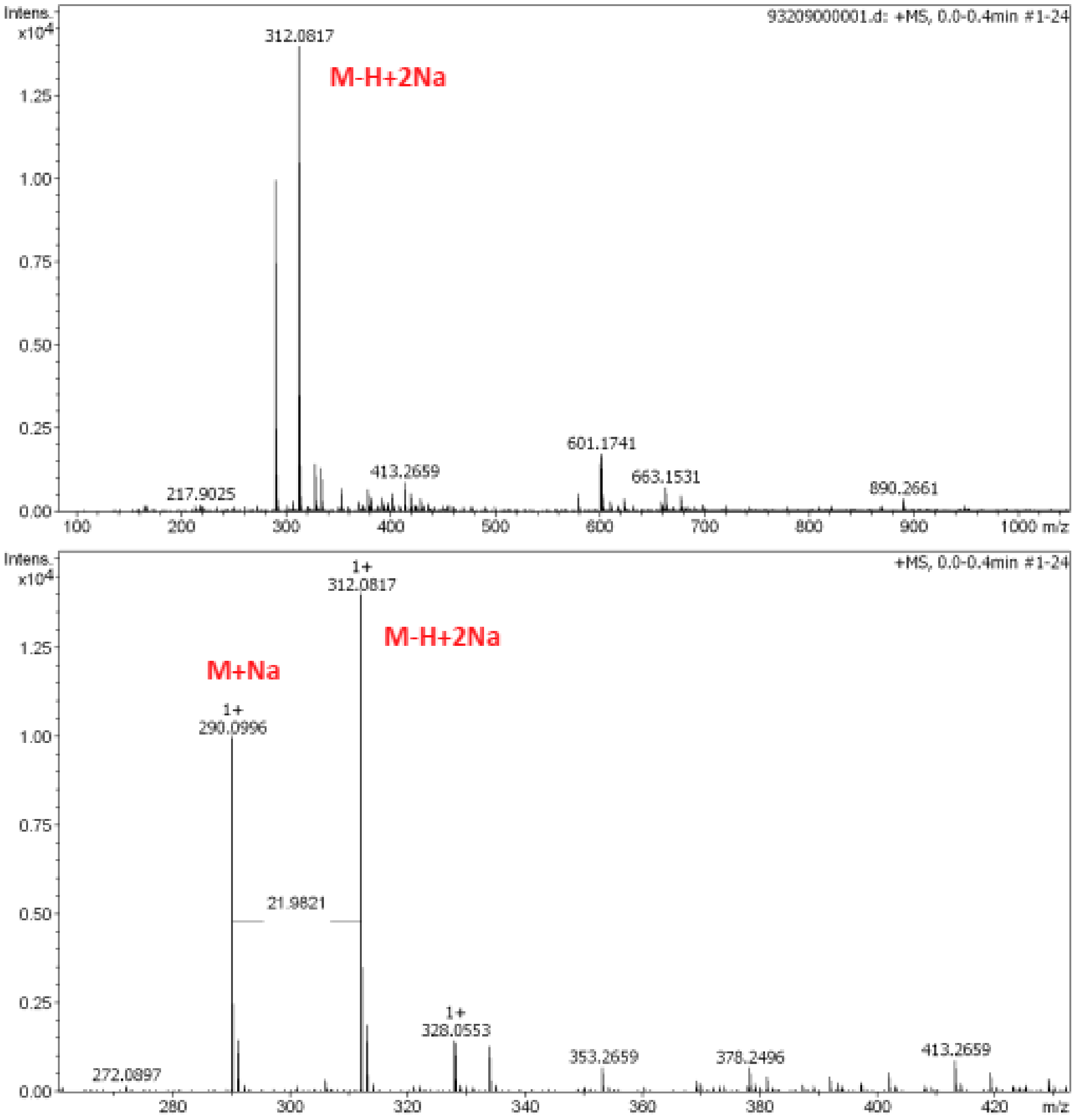

Figure S8. ESI mass spectrum of **5**., positive mode

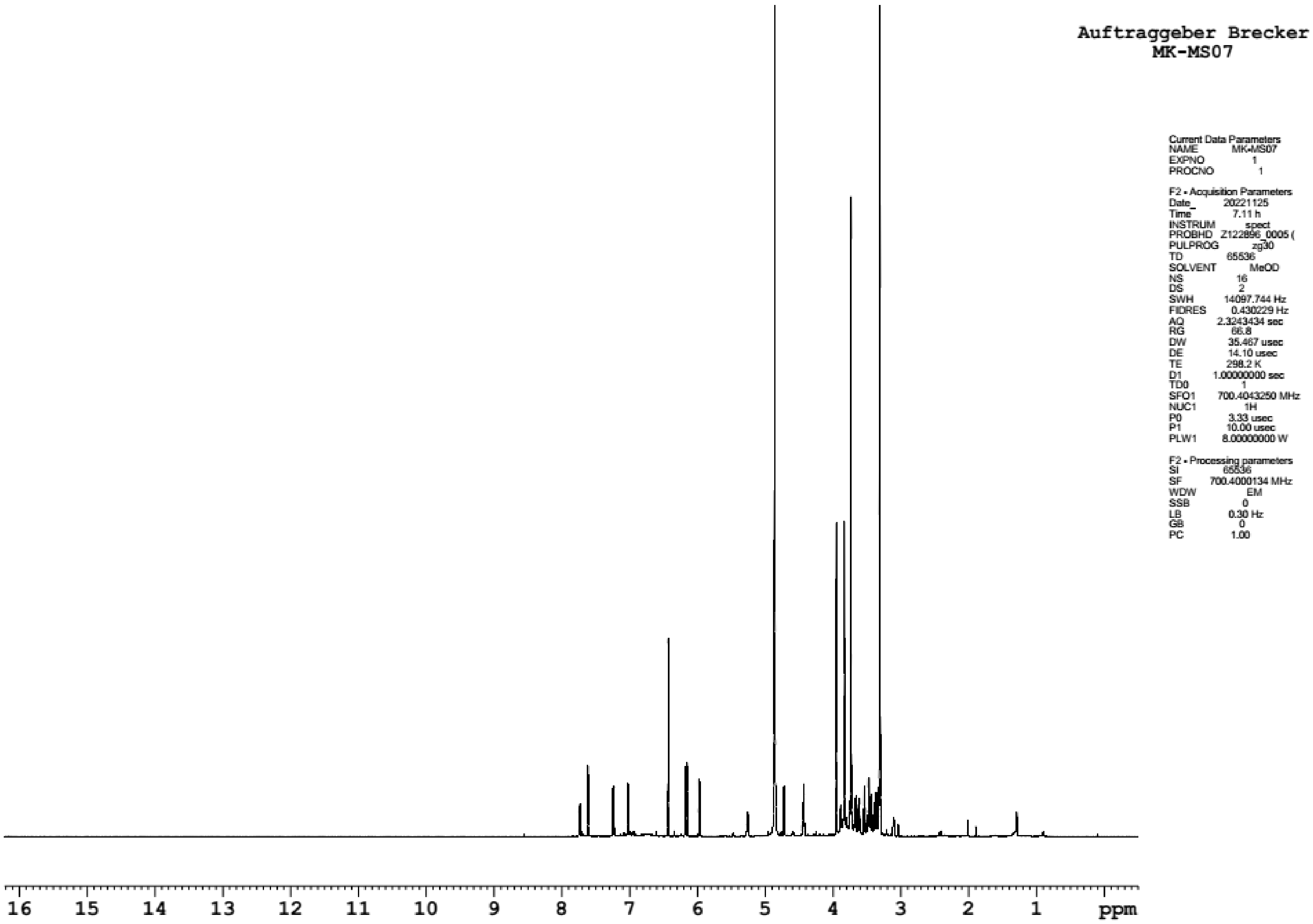

Figure S9. ^{1}H NMR spectrum of **8**.

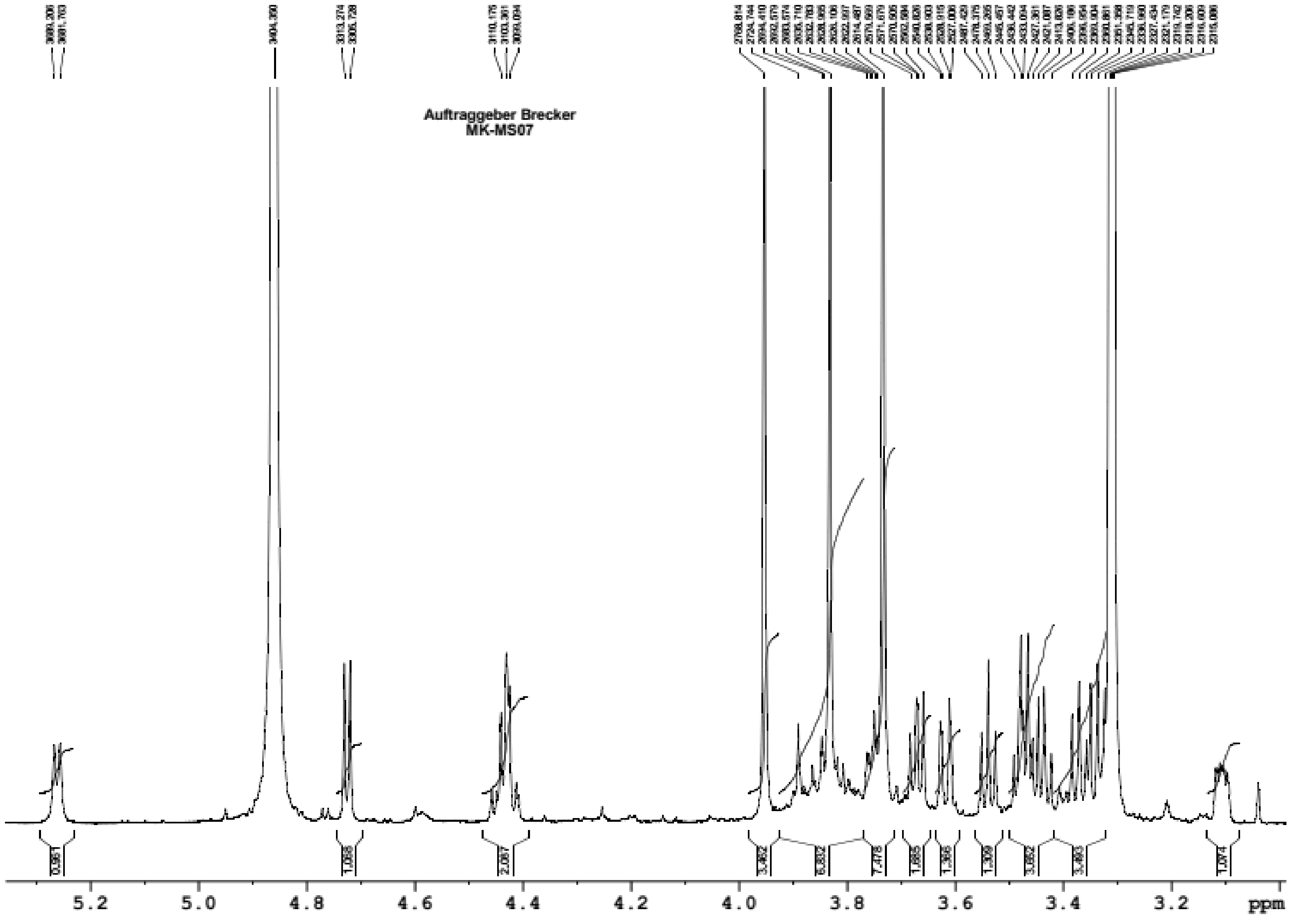

Figure S10. ^{1}H NMR spectrum of **8**, expansion

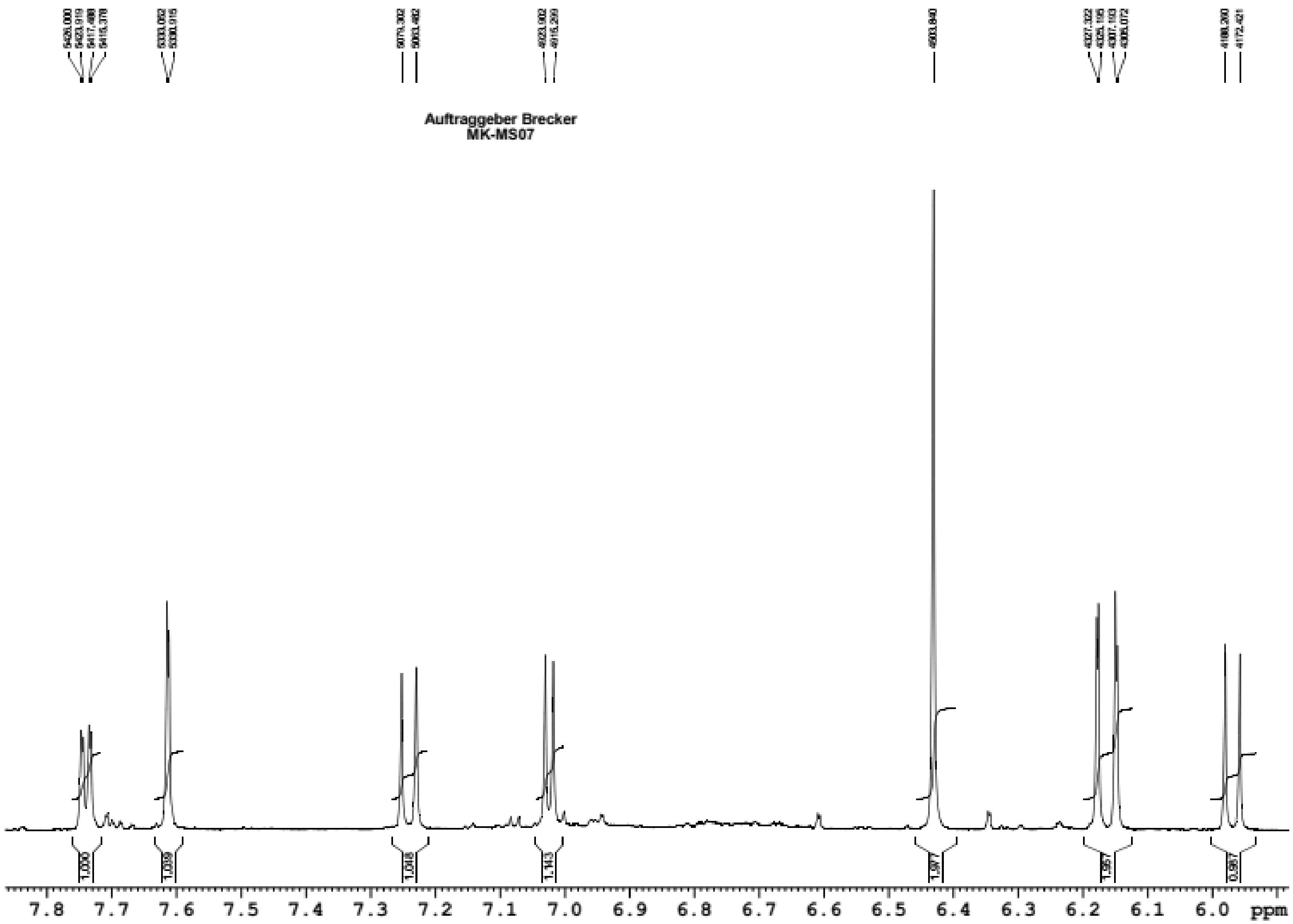

Figure S11. ^{1}H NMR spectrum of **8**, expansion

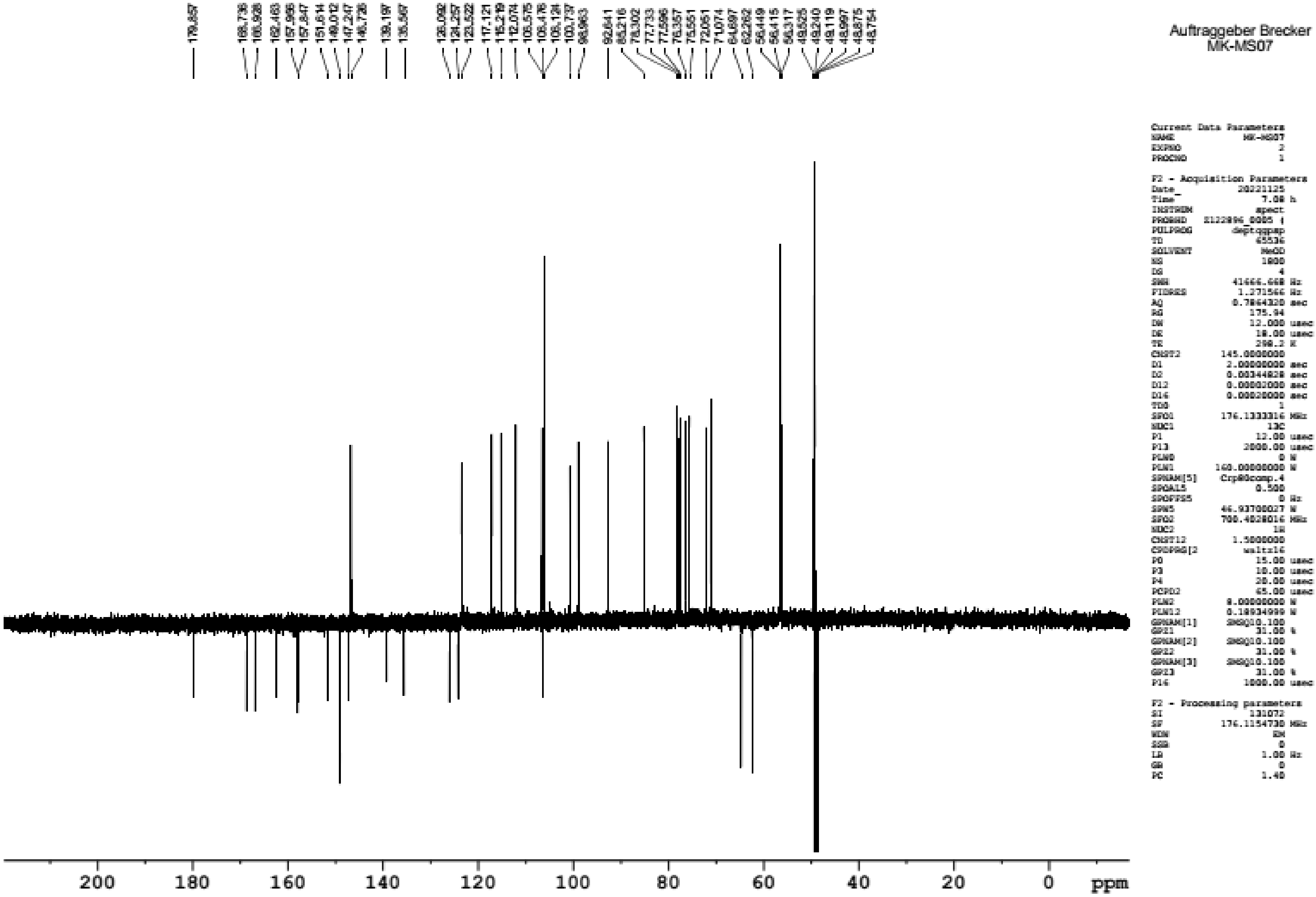

Figure S12. ^{13}C NMR spectrum of **8**.

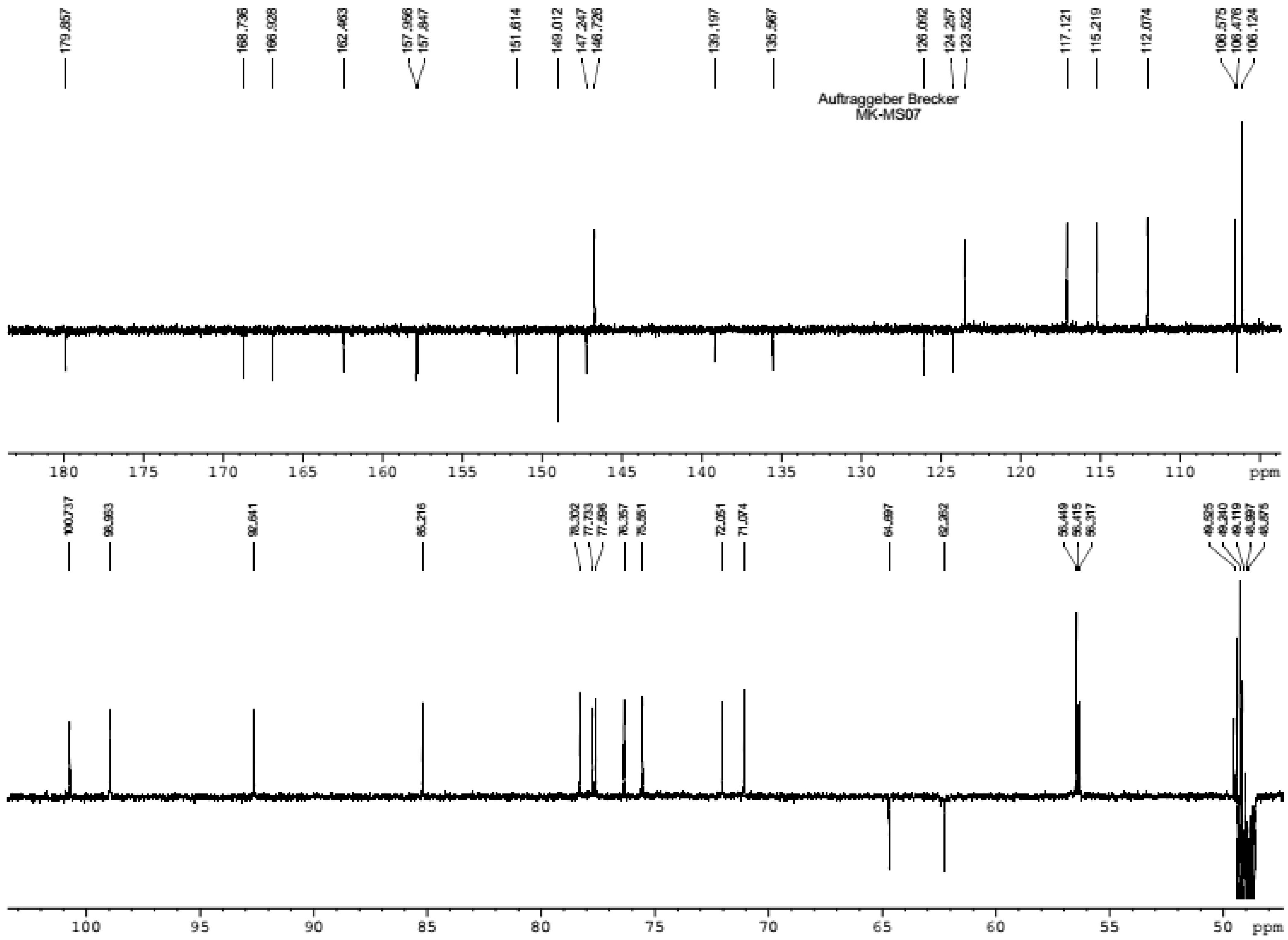

Figure S13. ^{13}C NMR spectrum of **8**, expansion.

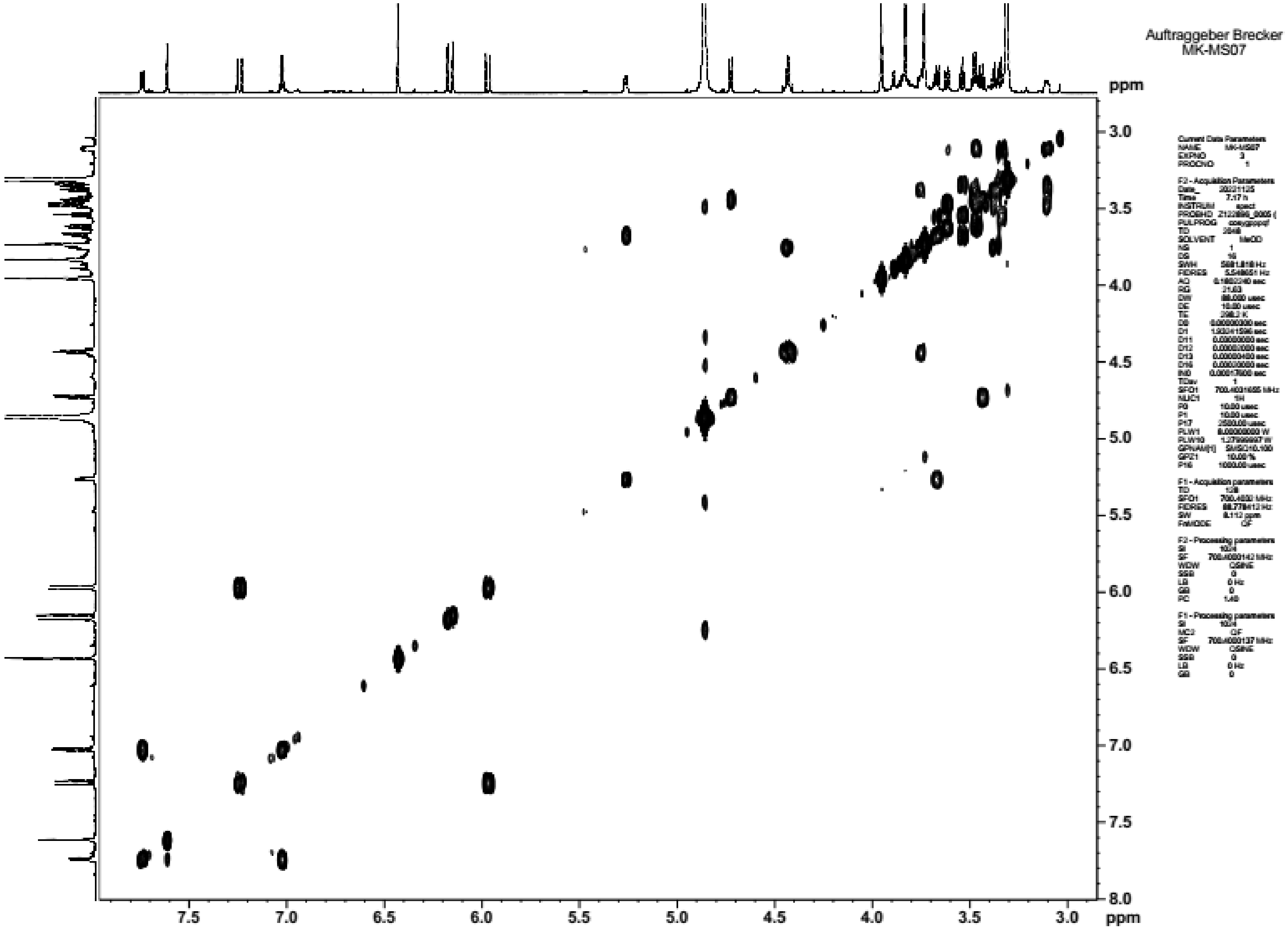

Figure S14. COSY NMR spectrum of **8**.

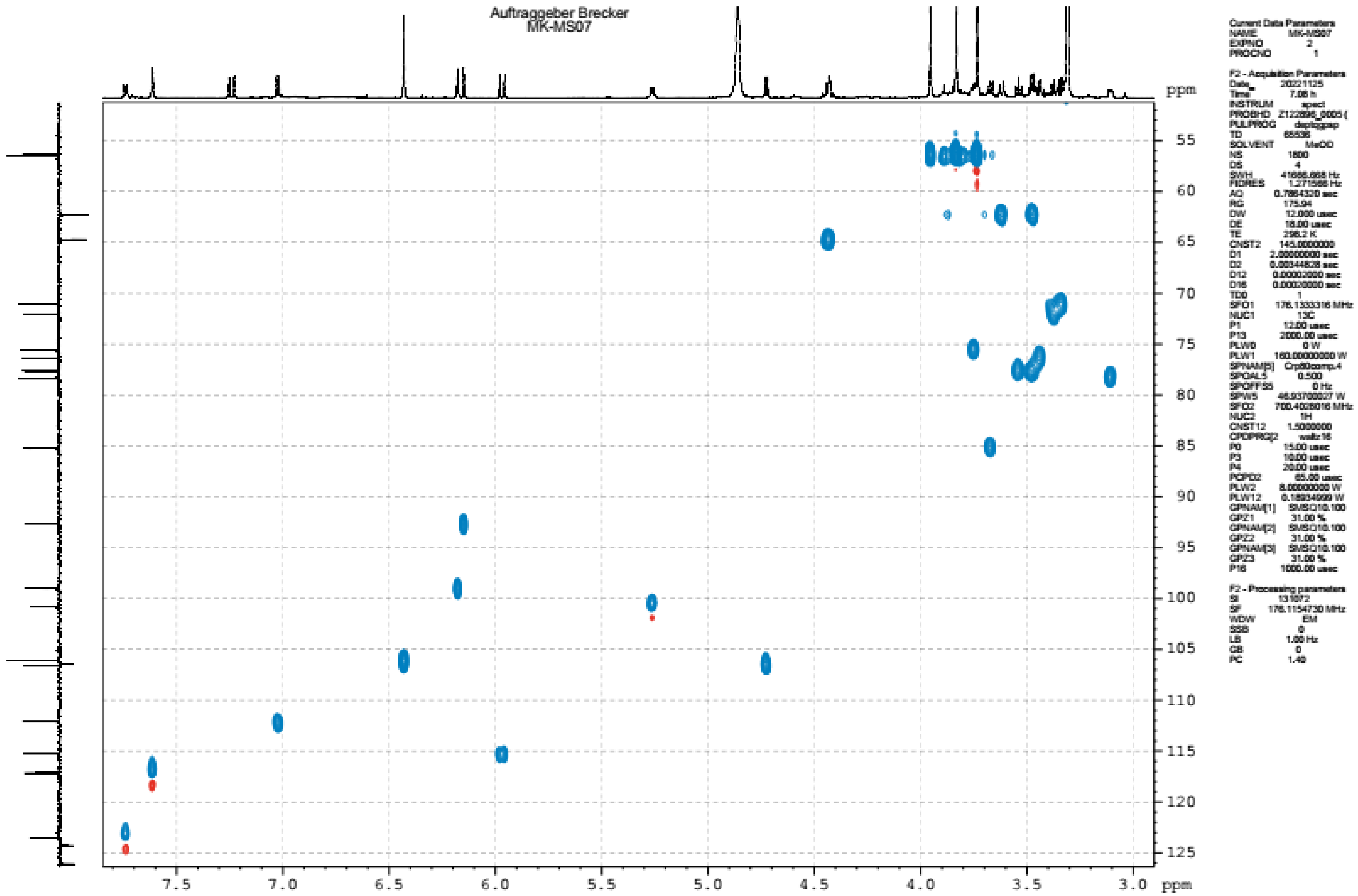

Figure S15. edited gs-HSQC NMR spectrum of **8**.

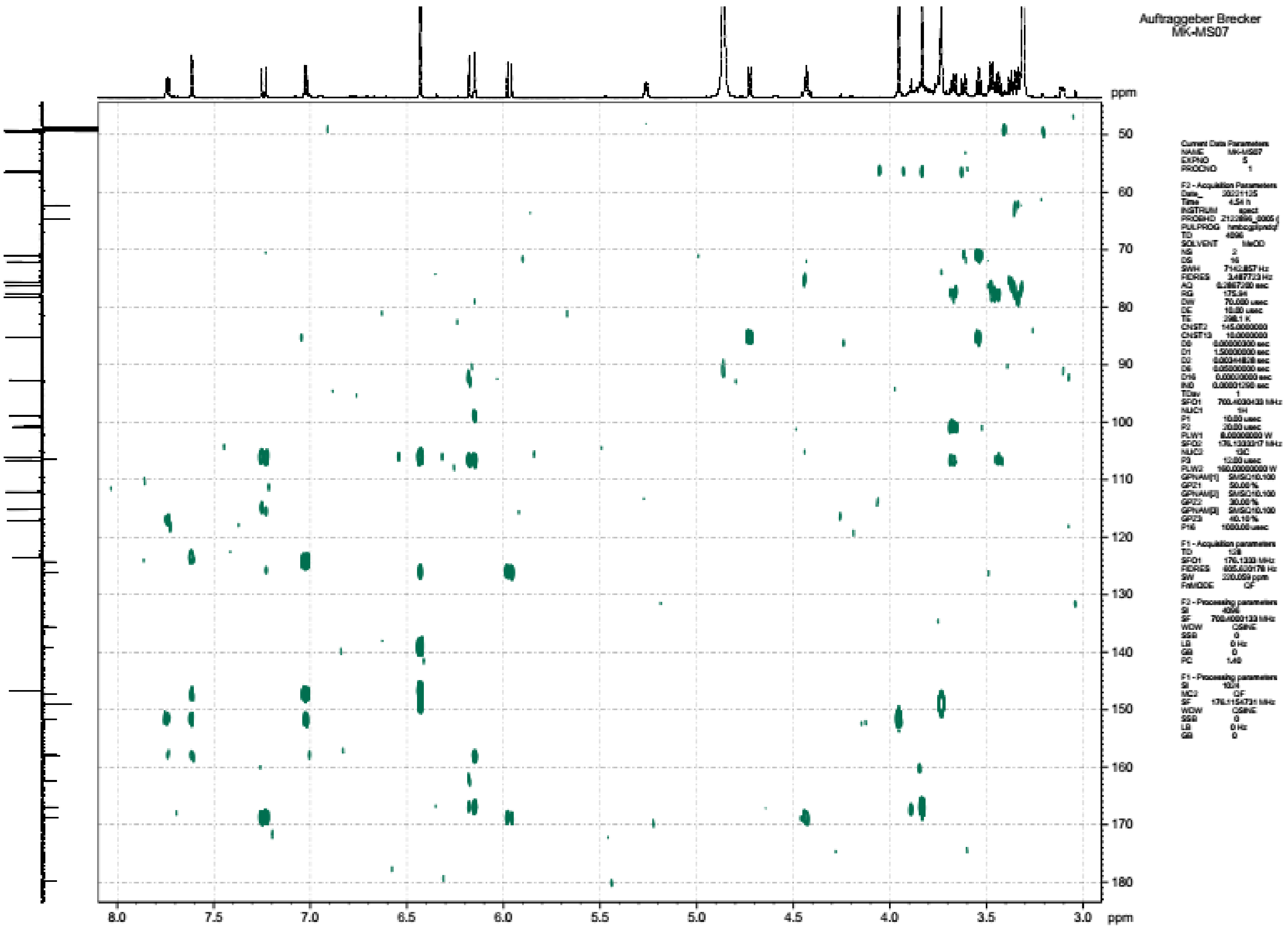

Figure S16. gs-HMBC NMR spectrum of **8**.

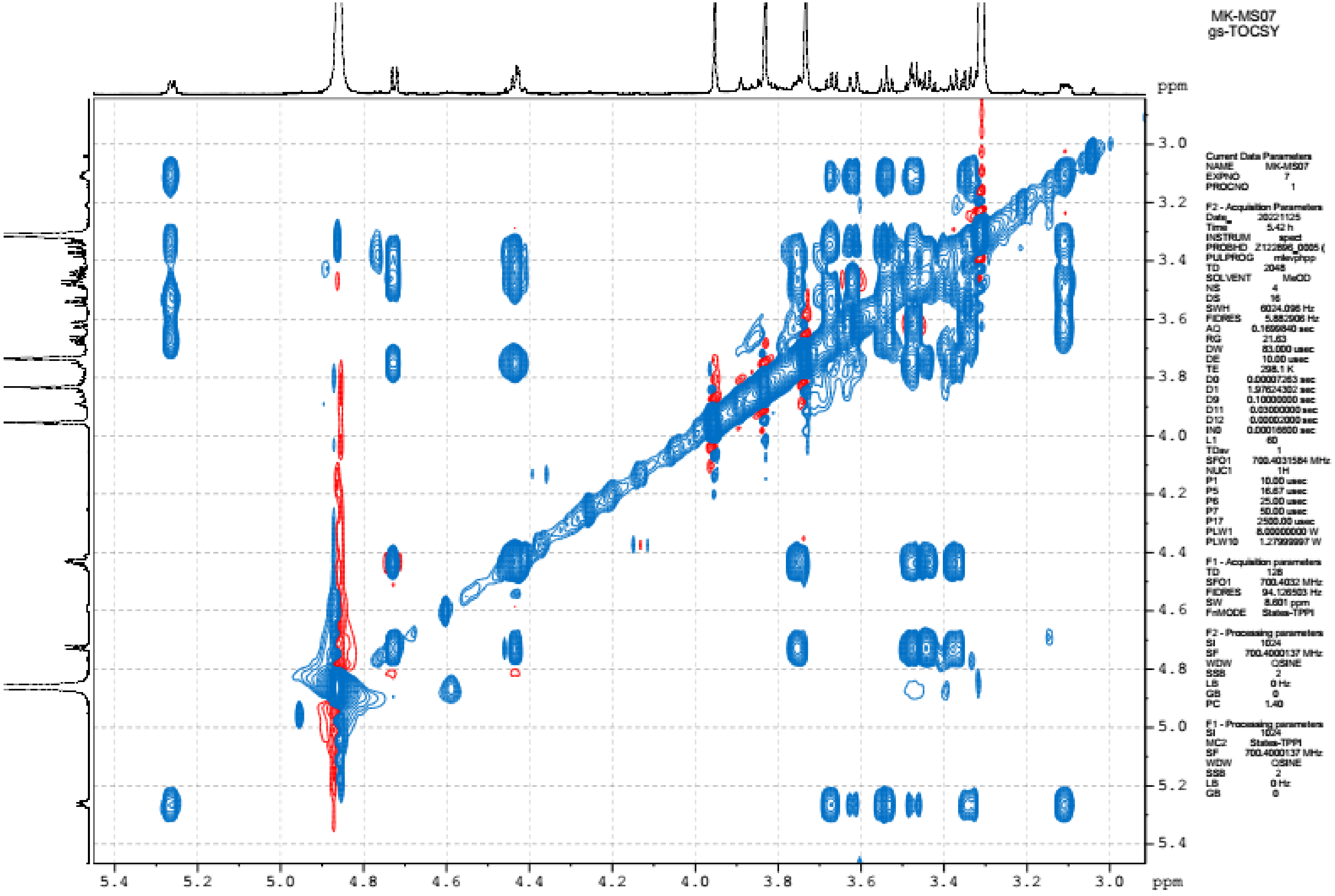

Figure S17. TOCSY NMR spectrum of **8**, carbohydrate region.

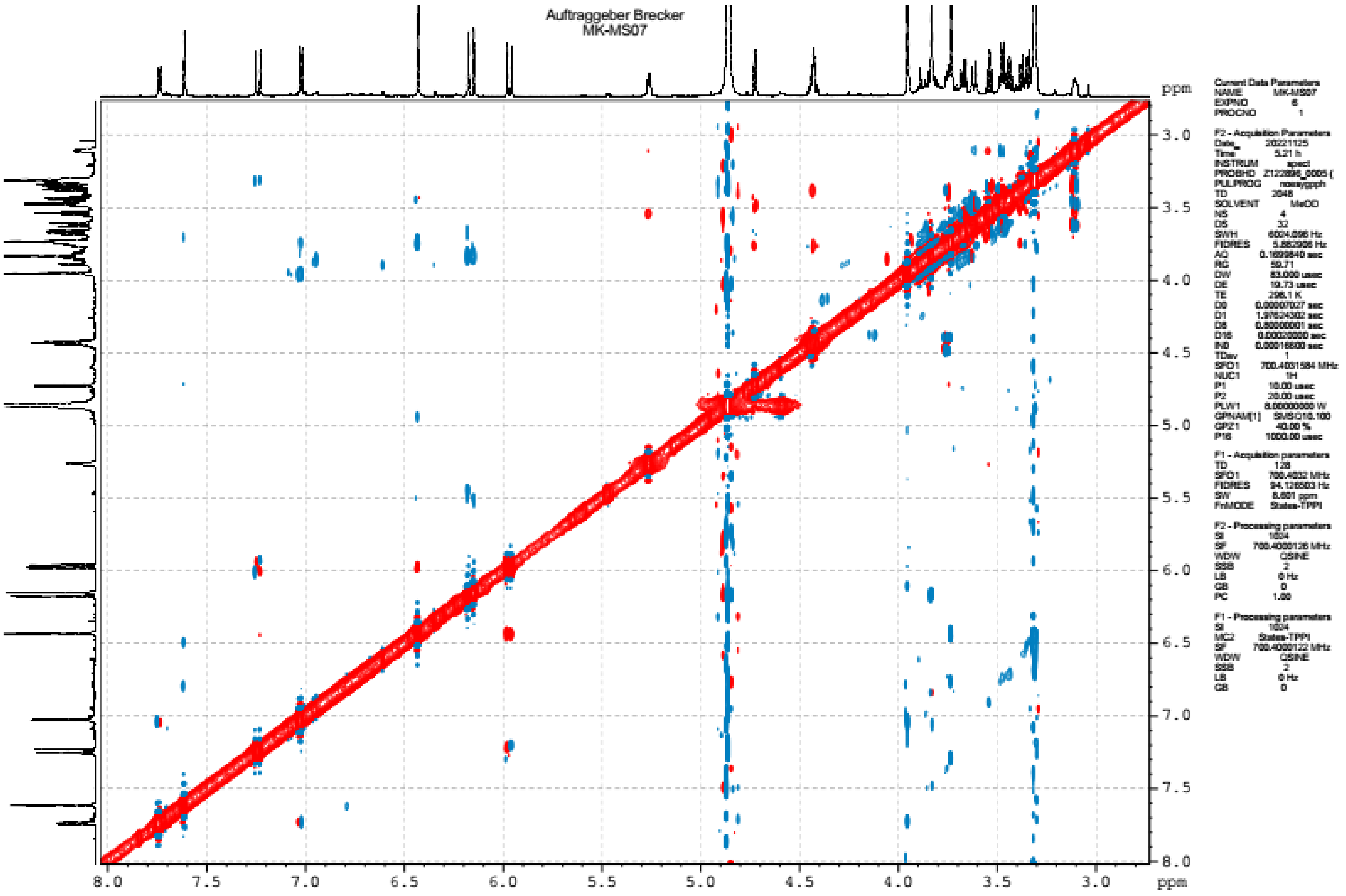

Figure S18. gs-NOESY NMR spectrum of **8**.

Generic Display Report

**Analysis Info**		Acquisition Date	11/24/2022 8:08:16 PM
Analysis Name	E:\Data\MS_MessService\93287000002.d		
Method	tune_low_MS_Service_neg_2022.m	Operator	msc
Sample Name	MK-MS07	Instrument	maXis
Comment	Schinnerl / Brecker / Botanik<br>Ergebnis +/- 5ppm<br>ACN / MeOH + 1% H2O		

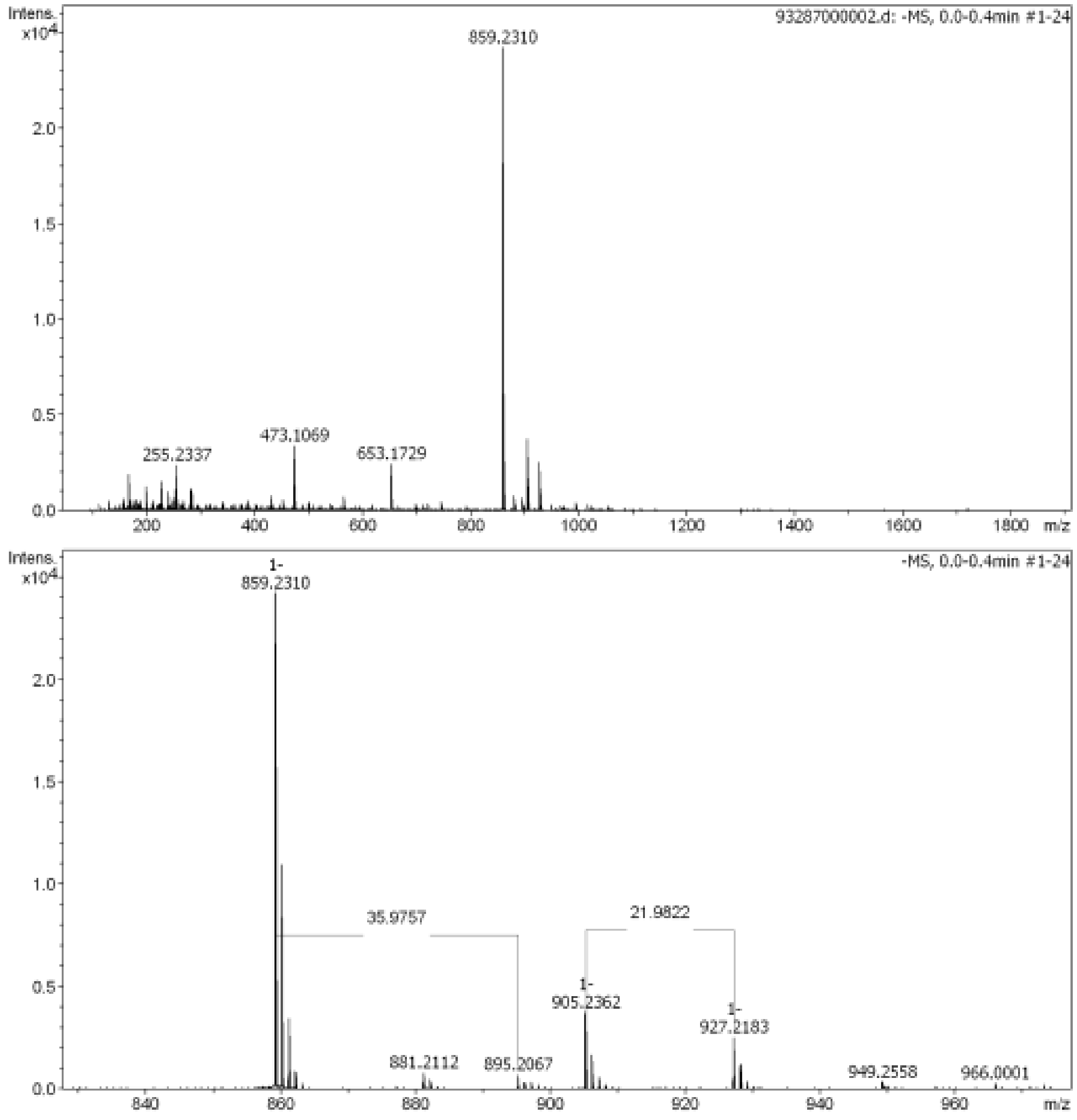

Figure S19. ESI mass spectrum of **8**, negative mode

Generic Display Report

Analysis Info

Analysis Name	E:\Data\MS_MessService\93287000001.d	Acquisition Date	11/24/2022 6:38:35 PM
Method	tune_low_MS_Service_11_22.m	Operator	msc
Sample Name	MK-MS07	Instrument	maXis
Comment	Schinnerl / Brecker / Botanik<br>Ergebnis +/- 5ppm<br>ACN / MeOH + 1% H2O		

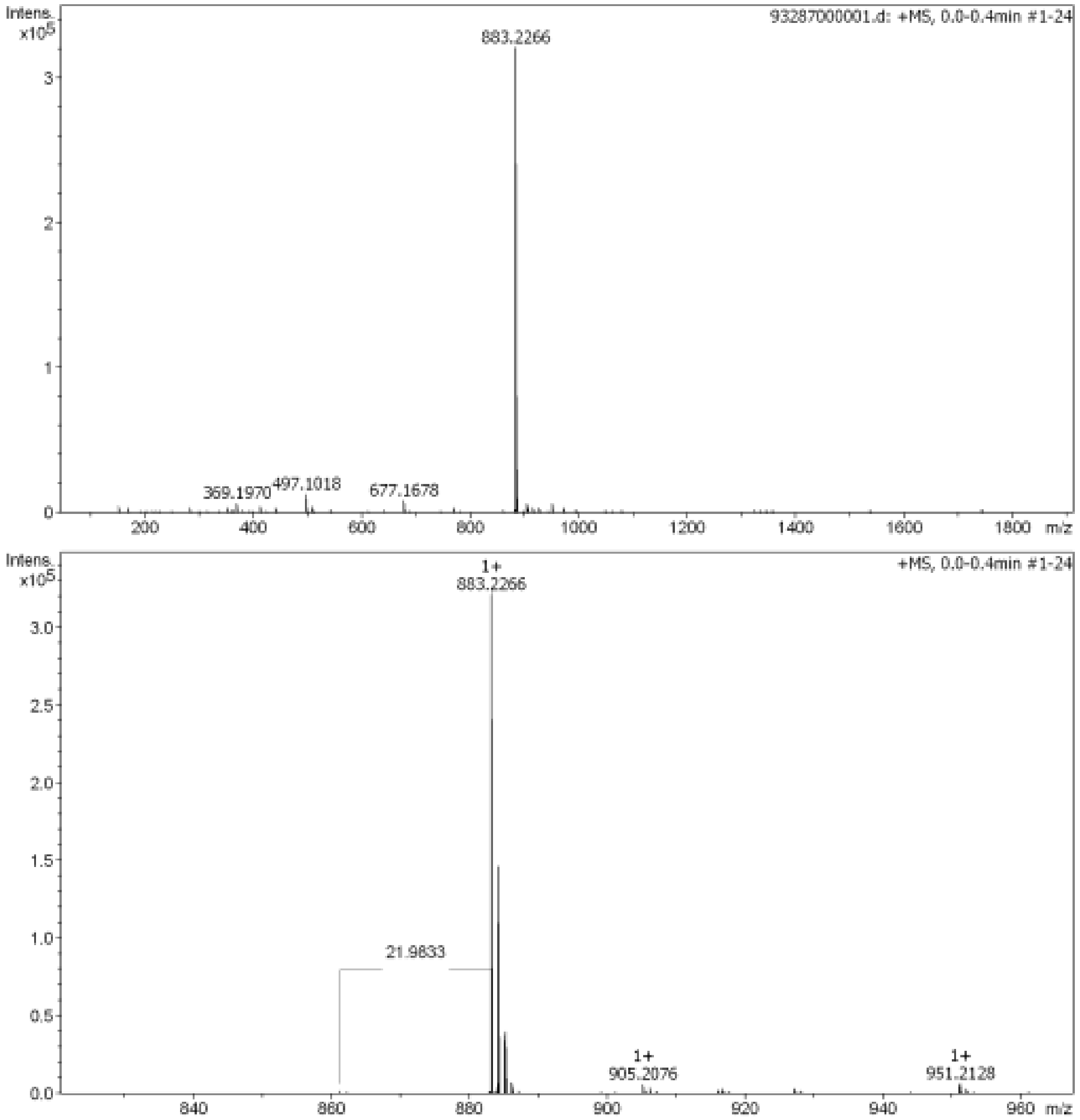

Figure S20. ESI mass spectrum of **8**, positive mode.

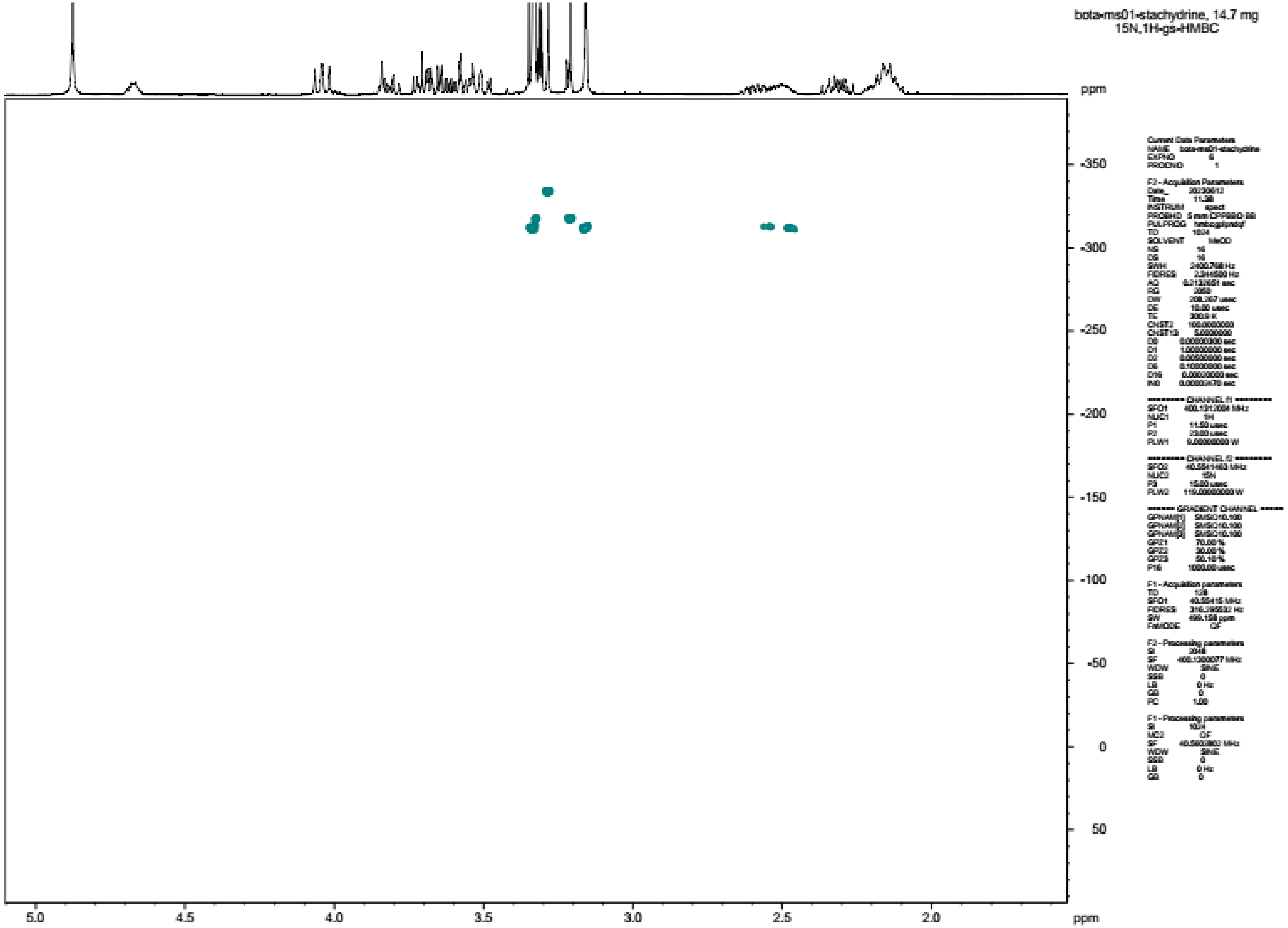

Figure S21. $^{1}H,^{15}N$-HMBC spectrum of a mixture of **9**, **10**, and betain.
